# Interferon-gamma release assay for the diagnosis of latent tuberculosis infection: A latent-class analysis

**DOI:** 10.1371/journal.pone.0188631

**Published:** 2017-11-28

**Authors:** Tan N. Doan, Damon P. Eisen, Morgan T. Rose, Andrew Slack, Grace Stearnes, Emma S. McBryde

**Affiliations:** 1 Department of Medicine at The Royal Melbourne Hospital, University of Melbourne, Melbourne, Victoria, Australia; 2 Australian Institute of Tropical Health and Medicine, James Cook University, Townsville, Queensland, Australia; 3 Institute of Health and Biomedical Innovation, Queensland University of Technology, Brisbane, Queensland, Australia; 4 College of Medicine and Dentistry, James Cook University, Townsville, Queensland, Australia; 5 Townsville Hospital and Health Service, Townsville, Queensland, Australia; 6 Department of Infectious Diseases, Alfred Health, Melbourne, Victoria, Australia; Chinese Academy of Medical Sciences and Peking Union Medical College, CHINA

## Abstract

**Background:**

Accurate diagnosis and subsequent treatment of latent tuberculosis infection (LTBI) is essential for TB elimination. However, the absence of a gold standard test for diagnosing LTBI makes assessment of the true prevalence of LTBI and the accuracy of diagnostic tests challenging. Bayesian latent class models can be used to make inferences about disease prevalence and the sensitivity and specificity of diagnostic tests using data on the concordance between tests. We performed the largest meta-analysis to date aiming to evaluate the performance of tuberculin skin test (TST) and interferon-gamma release assays (IGRAs) for LTBI diagnosis in various patient populations using Bayesian latent class modelling.

**Methods:**

Systematic search of PubMeb, Embase and African Index Medicus was conducted without date and language restrictions on September 11, 2017 to identify studies that compared the performance of TST and IGRAs for LTBI diagnosis. Two IGRA methods were considered: QuantiFERON-TB Gold In Tube (QFT-GIT) and T-SPOT.TB. Studies were included if they reported 2x2 agreement data between TST and QFT-GIT or T-SPOT.TB. A Bayesian latent class model was developed to estimate the sensitivity and specificity of TST and IGRAs in various populations, including immune-competent adults, immune-compromised adults and children. A TST cut-off value of 10 mm was used for immune-competent subjects and 5 mm for immune-compromised individuals.

**Findings:**

A total of 157 studies were included in the analysis. In immune-competent adults, the sensitivity of TST and QFT-GIT were estimated to be 84% (95% credible interval [CrI] 82–85%) and 52% (50–53%), respectively. The specificity of QFT-GIT was 97% (96–97%) in non-BCG-vaccinated and 93% (92–94%) in BCG-vaccinated immune-competent adults. The estimated figures for TST were 100% (99–100%) and 79% (76–82%), respectively. T-SPOT.TB has comparable specificity (97% for both tests) and better sensitivity (68% versus 52%) than QFT-GIT in immune-competent adults. In immune-compromised adults, both TST and QFT-GIT display low sensitivity but high specificity. QFT-GIT and TST are equally specific (98% for both tests) in non-BCG-vaccinated children; however, QFT-GIT is more specific than TST (98% versus 82%) in BCG-vaccinated group. TST is more sensitive than QFT-GIT (82% versus 73%) in children.

**Conclusions:**

This study is the first to assess the utility of TST and IGRAs for LTBI diagnosis in different population groups using all available data with Bayesian latent class modelling. Our results challenge the current beliefs about the performance of LTBI screening tests, and have important implications for LTBI screening policy and practice. We estimated that the performance of IGRAs is not as reliable as previously measured in the general population. However, IGRAs are not or minimally affected by BCG and should be the preferred tests in this setting. Adoption of IGRAs in settings where BCG is widely administered will allow for a more accurate identification and treatment of LTBI.

## Introduction

Reliable detection of latent tuberculosis infection (LTBI) is a priority as this will help direct appropriate use of limited resources for tuberculosis (TB) control. One-third of the world’s population have LTBI with 10% of these individuals eventually developing active TB [1]. The risk of progression from LTBI to active TB is considerably higher in the presence of predisposing factors such as immune-compromised conditions [2]. Treatment costs of TB, particularly multi-drug-resistant infection are high [3]. Cases with pulmonary TB disease are the source of ongoing transmission in the community.

Diagnosis of LTBI suffers from the absence of a gold standard test. The tuberculin skin test (TST) remains the most widely used principally due to its low cost. However, it is substantially affected by cross-reactivity with non-tuberculous mycobacterial proteins found in the Bacillus Calmette-Guérin (BCG) vaccine, causing false-positive test results [4]. Interferon-gamma release assays (IGRAs), including the commercially available assays QuantiFERON-TB Gold In Tube (QFT-GIT; Qiagen, Hilden, Germany), and the T-SPOT.TB (Oxford Immunotec, Oxfordshire, UK), are used as alternatives to TST in settings where higher test acquisition costs can be supported. IGRAs are thought to be more specific than TST as they measure interferon-gamma released by T-cells after stimulation with *Mycobacterium tuberculosis*-specific antigens absent in BCG and most non-tuberculosis mycobacteria [5].

The diagnostic performance of IGRAs for LTBI in clinical practice has been evaluated in a number of studies in immune-competent adults, which largely show that these tests have higher specificity than TST [6,7]. The data on the reliability of IGRAs for the diagnosis of LTBI in immune-compromised adults and children have not been resolved with certainty. Without a gold standard, the true prevalence of disease and accuracy of diagnostic tests are difficult to measure reliably. Many studies have instead compared the performance of IGRAs against TST by evaluating the agreement between these tests.

Bayesian latent class models can be used to make inferences about disease prevalence and the sensitivity and specificity of diagnostic tests using data on the concordance between tests [8–10]. This approach is based on the notion that the observed results of various imperfect diagnostic tests for the same disease are influenced by an underlying unobserved (i.e. latent) variable, the true disease status [8–10]. In this study, we used the Bayesian latent class modelling approach to evaluate the diagnostic performance of IGRAs (QFT-GIT and T-SPOT.TB) and TST for the diagnosis of LTBI in various population groups.

## Methods

### Search strategy and selection criteria

A systematic literature search of PubMed, Embase and African Index Medicus databases was conducted on September 11, 2017 to identify original studies that evaluated the concordance between TST and QFT-GIT or T-SPOT.TB for the diagnosis of LTBI in human subjects. The search included the following Medical Subject Headings (MeSH) terms or text key words: (tuberculin[mesh]) OR “TST” OR “Mantoux”) and (“interferon gamma release assay” OR “interferon gamma assay” OR “QuantiFero*” OR “IGRA” OR “T-SPO*” OR “TSPO*” OR “Elispot” OR CFP10 OR ESAT6) and (tuberculosis[mesh]). No restrictions on date, language, or type of studies were applied. The full search strategy is described in S1 Text. Secondary searching of the reference lists of relevant articles and reviews was also performed for saturation. Titles and abstracts were screened by three authors (TD, AS, and GS) to remove articles that were not relevant to our study. After this initial screening, full-texts of potentially relevant studies were obtained and reviewed independently by at least two of the authors (TD, DE, AS, and GS). Articles were included in this study if they met the following data criteria: 2x2 agreement tables or sufficient information that allowed the construction of such tables between TST and QFT-GIT or T-SPOT.TB; used a TST cut-off value of 5 mm or 10 mm; included IGRAs that were commercial versions using a mixture of the synthetic peptides ESAT-6 and CFP-10; and that the tests were used for the diagnosis of LTBI. This study was reported in accordance with the PRISMA Statement [11]. The review protocol was registered with the International prospective register of systematic reviews (PROSPERO) (CRD42017060705).

### Data synthesis and analysis

Data from each eligible study were extracted by two independent reviewers. Discrepancies between the two reviewers were resolved by consensus or by consultation with a third reviewer (DE) if consensus could not be reached. The following variables were extracted: year of publication, country of origin, population group, BCG vaccination rate, TST cut-off value, methods of IGRAs, age range and mean/median where available, proportion of participants on immunosuppressive therapy, and 2x2 test agreement data (TST+/IGRA+, TST+/IGRA-, TST-/IGRA+, TST-/IGRA-). If separate agreement tables were available for different subgroups of patients, these data were included separately [6]. Authors were contacted for further information where appropriate. The QUADAS-2 checklist for the quality assessment of diagnostic accuracy studies was used for quality assessment of the included studies [12]. A description of the QUADAS-2 items can be found in S2 Text.

The primary outcome was the diagnostic performance, i.e. sensitivity, specificity, positive predictive value and negative predictive value, of TST, QFT-GIT and T-SPOT.TB in immune-competent adults aged 15 years or above. For studies to be included in this primary analysis, the prevalence of immune-compromised conditions had to be less than 5% [6]. Subgroup analyses investigating the diagnostic performance of TST and QFT-GIT were performed on immune-competent children (≤ 14 years of age) and immune-compromised adults. Subgroup analyses on these population groups were not performed with T-SPOT.TB due to insufficient data. In accordance with international guidelines [13–15] and real-life clinical practice, we used a TST cut-off value of 10 mm for immune-competent subjects and 5 mm for immune-compromised individuals. We allowed for factors that could potentially lead to variability of diagnostic test performance between studies including BCG vaccination rate and immune status.

### Bayesian latent class model

We developed a Bayesian latent class model to describe the observed 2x2 data to estimate the true prevalence (*π*) of LTBI in the population, and the sensitivity (*S*_1_, *S*_2_) and specificity (*C*_1_, *C*_2_) of TST (test 1, *T*_1_) and IGRA (test 2, *T*_2_). Let *D* be the unknown (latent) true disease status, the prevalence, sensitivity and specificity can be formally expressed as follows:

$$\pi =P(D+),S_{1}=P(T_{1}+|D+),S_{2}=P(T_{2}+|D+),C_{1}=P(T_{1}-|D-),\;and\;C_{2}=P(T_{2}-|D-).$$ (1)

The observed data follow a multinomial distribution where each probability of the four combinations of the results of the two tests can be expressed in terms of *π*, *S*_1_, *S*_2_, *C*_1_ and *C*_2_ as follows:

$$\begin{array}{l} P(T_{1}+,\;T_{2}+)=\pi S_{1}S_{2}+(1-\pi )(1-C_{1})(1-C_{2});\\ P(T_{1}+,\;T_{2}-)=\pi S_{1}(1-S_{2})+(1-\pi )(1-C_{1})C_{2};\\ P(T_{1}-,\;T_{2}+)=\pi (1-S_{1})S_{2}+(1-\pi )C_{1}(1-C_{2});\\ P(T_{1}-,\;T_{2}-)=\pi (1-S_{1})(1-S_{2})+(1-\pi )C_{1}C_{2}. \end{array}$$ (2)

In the latent class model in Eq 2, *π*, *S*_1_, *S*_2_, *C*_1_ and *C*_2_ were the unknown model parameters to be estimated. A Bayesian approach was used to make inferences about these unknown parameters. This approach combines the observed data, i.e. 2x2 table, and prior knowledge about the parameters formally expressed as a prior probability distribution, to obtain a posterior probability distribution of the unknown parameters. We assumed a beta(*α*,*β*) distribution for the priors of the sensitivity and specificity. Beta distribution was chosen because its region of positive density ranges from 0 to 1, matching the range of these parameters [8]. It also has the advantage of being flexible, allowing a wide variety of the shapes of the distribution to be determined by selecting different choices of *α* and *β* [8]. The *α* and *β* parameters of the beta distributions of the sensitivity and specificity of *T*_1_ and *T*_2_ were determined by equating the midpoint of the range reported in the literature to the mean (*μ*) of the beta distribution, and equating one quarter of the range to the standard deviation (*δ*) of the beta distribution [10]. The mean, standard deviation and the parameters of a beta distribution were given by the following equations:

$$\begin{array}{l} \mu =\frac{\alpha }{\alpha +\beta };\\ \delta =\sqrt{\frac{\alpha \beta }{{\left(\alpha +\beta \right)}^{2}(\alpha +\beta +1)}};\\ \alpha =-\frac{\mu (\delta^{2}+\mu^{2}-\mu )}{\delta^{2}};\\ \beta =\frac{\left(\mu -1)(\delta^{2}+\mu^{2}-\mu \right)}{\delta^{2}}. \end{array}$$ (3)

For TST (*T*_1_), the sensitivity reported in the literature ranged from 57% to 95% [16,17], while the specificity ranged from 55% to 100% [18,19]. Using Eq 3, these corresponded to beta(14.6, 4.6) and beta(9.9, 2.88) for *S*_1_ and *C*_1_, respectively. The sensitivity of IGRAs reported in the literature ranged from 55% to 93% [18,20], and their specificity ranged from 89% to 100% [21,22]. These were converted into beta(15.04, 5.28) and beta(64, 3.7) for *S*_2_ and *C*_2_, respectively. A uniform(0, 0.9) was used for the priors of LTBI prevalence (*π*), knowing that the highest prevalence rate reported in the literature was 90% [23]. This distribution assigns equal weights to all possible values from 0 to 0.9 to allow LTBI prevalence to vary freely within this range among studies (i.e. populations). A separate estimate of prevalence for each population was performed.

We also estimated the effect of BCG on the specificity of the tests as follows:

$$C_{1i}=pE_{BCG}+(1-p)C_{1};$$ (4)

where *C*_1*i*_ is the specificity of a test in the current (*i*^th^) population, *p* is the proportion of individuals in that population who is vaccinated, and *E*_*BCG*_ is the effect of BCG on the specificity of the test in that population.

Positive predictive value (PPV) and negative predictive value (NPV) were also estimated using the following formulae. S3 Text describes how these formulae were derived.

$$\begin{array}{l} PPV=P(D+|T+)=\frac{S\pi }{S\pi +(1-C)(1-\pi )};\\ NPV=P(D-|T-)=\frac{C(1-\pi )}{\left(1-S)\pi +C(1-\pi \right)}. \end{array}$$ (5)

Bayesian inferences with the Gibbs sampler algorithm was used to estimate the model parameters. For each parameter, three Markov chains were constructed, each chain with different initial values. Convergence of the Markov chains was assessed by visual inspection of the density plots of parameter estimates and by examining the Gelman-Rubin statistics [24]. A Gelman-Rubin value of less than 1.1 was considered convergence [24]. We ran each chain with 70,000 iterations and a burn-in period of 10,000. For each parameter, median estimates and their 95% credible interval (CrI) were reported. The log-odds ratio check (LORC) method was used for assessment of conditional independence between the two test observations [25]. Briefly, the LORC investigates how well a model describes a particular dataset by comparing the empirical pairwise log-odds ratios with the pairwise predicted log-odds ratios [25]. The difference between the observed and expected log-odds ratios is expressed by a z-score. A z-score within the ±1.96 range indicates that the assumption of conditional independence is valid [25]. All analyses were performed in WinBUGS (version 1.4, Imperial College & Medical Research Council, UK). As this study used data from published literature, ethics approval was not required.

## Results

**Fig 1:**
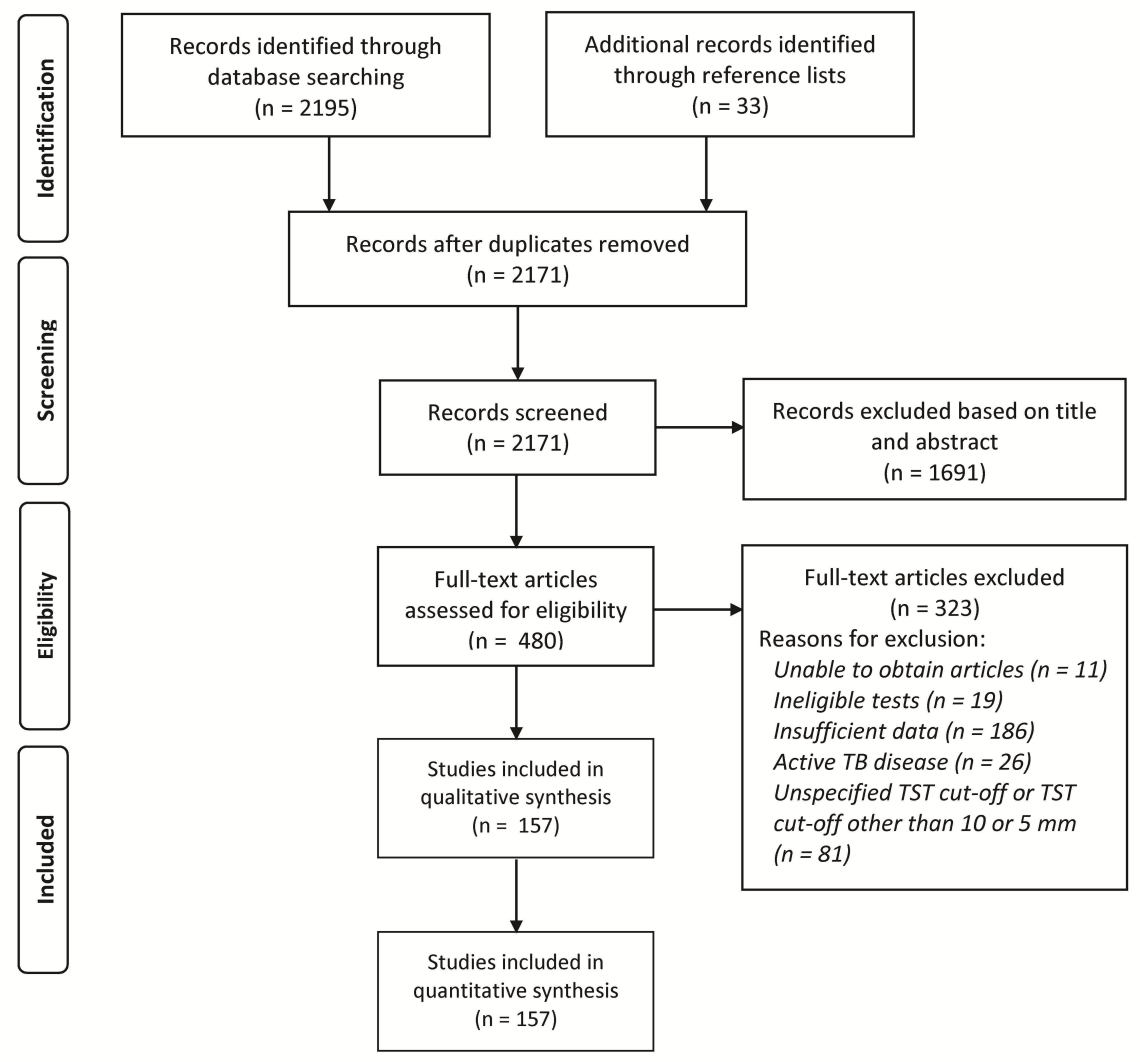
Flowchart of study selection. TB, tuberculosis; TST, tuberculin skin test.

A total of 2,195 articles were identified from the initial searches. After assessment of titles and abstracts, 480 articles were assessed as potentially relevant and their full-texts were reviewed. Of these, 157 articles met the a priori inclusion criteria [26–182]. These studies comprised 170 agreement tables. The earliest and latest years of publication were 2006 and 2017, respectively. Of the included studies, four were published in languages other than English (one Polish, three Spanish); however, the full-texts of these studies were already translated into English by the journal. Fig 1 outlines how the final sample size was reached.

The characteristics of the included studies are shown in Table 1. Eighty seven percent (137/157) of the included studies reported rates of BCG vaccination. The majority (132/157, 84%) of the studies were conducted in adults (≥15 years of age). Twenty five percent (39/157) of the studies were conducted in patients selected because of altered immunity due to the presence of HIV/AIDS, solid organ transplantation, stem cell transplantation, immune-mediated inflammatory diseases, end-stage kidney disease or malignancy. QFT-GIT was the most common IGRA, used in 87% (137/157) of the included studies. T-SPOT.TB was used in 15/157 studies; all of which included only immune-competent adults. The remaining studies (5/150) used both methods.

**Table 1:** Characteristics of the included studies.

Reference	Country	Population	Age range (years)	BCG rate (%)	2x2 data*
**Immune-competent,** **IGRA = QFT-GIT**					
Diel et al. (2006) [26]	Germany	Contacts	Any age	50.8	25, 39, 6, 239
Nakaoka et al. (2006) [27]	Nigeria	Contacts	1–14	90	40, 14, 8, 93
Tsiouris et al. (2006) [28]	South Africa	Students	5–15	72.3	51, 29, 10, 94
Adetifa et al. (2007) [29]	Gambia	Contacts	≥15	43	69, 16, 33, 57
Arend et al. (2007) [30]	Netherlands	Unvaccinated	≥17	0	74, 186, 7, 518
Dogra et al. (2007) [31]	India	Contacts	1–12	82	8, 2, 3, 92
Franken et al. (2007) [32]	Netherlands	Military personnel	≥18	12.6	19, 120, 2, 535
Silverman et al. (2007) [33]	Canada	Contacts	≥18	100	3, 10, 0, 9
Chun et al. (2008) [34]	Korea	Contacts	≤13	100	9, 12, 1, 47
		Healthy controls	≤14	100	1, 41, 0, 23
Mirtskhulava et al. (2008) [35]	Georgia	HCW	18–74	92	133, 44, 26, 62
Petrucci et al. (2008) [36]	Nepal	Contacts	≤15	84.9	65, 9, 5, 58
	Brazil	Contacts	≤15	84.9	33, 2, 12, 63
Baker et al. (2009) [37]	USA	Refugees	1–81	NR	85, 23, 20, 67
Bianchi et al. (2009) [38]	Italy	Contacts, Immigrants	≤16	51.5	33, 21, 27, 253
Fox et al. (2009) [39]	Israel	HCW	≥18	34	9, 22, 8, 52
Herrmann et al. (2009) [40]	France	HCW	24–53	100	4, 9, 2, 4
Kik et al. (2009) [41]	Netherlands	Contacts	≥16	NR	142, 97, 10, 33
Kim et al. (2009) [42]	Korea	Immune-competent	19–98	100	17, 8, 7, 53
Lien et al. (2009) [43]	Vietnam	HCW	20–58	32	114, 49, 21, 71
Lighter et al. (2009) [44]	USA	Mixed	≤18	36	27, 88, 4, 85
Machado et al. (2009) [45]	Brazil	Contacts	Any age	76	100, 44, 17, 94
Ringshausen et al. (2009) [46]	Germany	HCW	20–62	51	7, 22, 6, 108
Saracino et al. (2009) [47]	Italy	Immigrants	Any age	NR	49, 23, 58, 149
Torres Costa et al. (2009) [48]	Portugal	HCW	≥16	100	371, 532, 26, 289
Tripodi et al. (2009) [49]	France	HCW	20–60	100	23, 74, 5, 46
Vinton et al. (2009) [50]	Australia	HCW	20–66	78	16, 98, 5, 222
Zhao et al. (2009) [51]	USA	HCW	≥18	NR	10, 10, 0, 20
Adetifa et al. (2010) [52]	Gambia	Contacts	0.5–14	59	43, 14, 29, 127
Costa et al. (2010) [53]	Portugal	HCW	≥16	100	525, 792, 33, 332
Grare et al. (2010) [54]	France	Contacts	≥18	45.4	5, 10, 0, 22
Huang et al. (2010) [55]	Taiwan	Contacts	Any age	89	12, 24, 3, 39
Jong Lee et al. (2010) [56]	Korea	HCW	22–53	100	10, 21, 9, 42
Katsenos et al. (2010) [57]	Greece	Army recruits	18–35	100	11, 85, 2, 31
Lee et al. (2010) [58]	Korea	Contacts	16–70	67.2	97, 29, 11, 48
Torres Costa et al. (2010) [59]	Portugal	HCW	≥18	63.7	525, 792, 33, 332
Thomas et al. (2010) [60]	Bangladesh	Mixed	11–15.3	79	72, 16, 35, 105
Tsolia et al. (2010) [61]	Greece	Mixed	≥15	NR	58, 70, 4, 16
Caglayan et al. (2011) [62]	Turkey	HCW	Any age	87	33, 32, 1, 12
Diel et al. (2011) [63]	Germany	Contacts	1–62	52	138, 104, 60, 652
Kasambira et al. (2011) [64]	South Africa	Contacts	≤16	95	48, 7, 27, 154
Kus et al. (2011) [65]	Poland	Healthy	≥18	100	85, 140, 41, 186
Legesse et al. (2011) [66]	Ethiopia	General	18–70	17.4	151, 16, 76, 28
Moon et al. (2011) [67]	Korea	HCW	22–67	100	18, 34, 14, 90
Moyo et al. (2011) [68]	South Africa	Contacts	≤3	100	57, 13, 11, 295
Pavic et al. (2011) [69]	Croatia	Contacts	0–5	100	14, 11, 4, 112
Rafiza et al. (2011) [70]	Malaysia	HCW	19–56	99.7	11, 45, 2, 37
Shanaube et al. (2011) [71]	Zambia, South Africa	Contacts	≥15	NR	577, 148, 570, 508
Talebi-Taher et al. (2011) [72]	Iran	HCW	23–59	100	14, 91, 3, 92
Torres Costa et al. (2011) [73]	Portugal	HCW	≥18	68.2	850, 1252, 103, 679
Torres Costa et al. (2011) [74]	Portugal	HCW	≥16	98.6	153, 344, 8, 67
Weinfurter et al. (2011) [75]	USA	Mixed	≥13	36	167, 155, 64, 1267
Yassin et al. (2011) [76]	Ethiopia	Contacts	≥15	52	87, 39, 24, 59
		Healthy controls	≥15	52	6, 10, 12, 86
Bergot et al. (2012) [77]	France	Contacts	12–97	20.4	28, 50, 7, 62
Di Renzi et al. (2012) [78]	Italy	Staff of homeless shelter	25–71	6.5	22, 0, 2, 27
		Healthy controls	≥18	66	16, 12, 3, 10
He et al. (2012) [79]	Mongolia	HCW	18–72	26	350, 89, 288, 190
Jeong et al. (2012) [80]	Korea	X-ray healed TB	36–88	42.6	79, 10, 48, 26
Jo et al. (2012) [81]	Korea	Contacts	Any age	78.2	34, 14, 20, 33
Jung da et al. (2012) [82]	Korea	Medical students	≥18	86.3	6, 17, 2, 128
Larcher et al. (2012) [83]	Italy	HCW	19–64	38	57, 103, 24, 365
Onur et al. (2012) [84]	Turkey	Outpatient paediatric clinic	≤14	87.6	33, 18, 4, 36
Pattnaik et al. (2012) [85]	India	Contacts	≥15	40.7	64, 24, 1, 11
Zwerling et al. (2012) [86]	Canada	HCW	≥18	36.1	7, 15, 17, 348
Jo et al. (2013) [87]	Korea	HCW	≥20	81	54, 127, 31, 281
Serrano-Escobedo et al. (2013) [88]	Mexico	Contacts	≥18	87	31, 11, 20, 61
Whitaker et al. (2013) [89]	Georgia	HCW	≥18	89	68, 38, 9, 39
Zwerling et al. (2013) [90]	Canada	HCW	≥18	61.6	3, 10, 10, 234
Alvarez et al. (2014) [91]	Canada	High risk groups	Any age	73	46, 40, 4, 166
Charisis et al. (2014) [92]	Greece	HCW	≥20	68	30, 179, 2, 32
de Souza et al. (2014) [93]	Brazil	HCW	≥18	86.4	114, 138, 58, 322
Erkens et al. (2014) [94]	Netherlands	Mixed	Any age	40	870, 1777, 66, 639
Garazzino et al. (2014) [95]	Italy	General	≤2	NR	0, 10, 9, 463
Garcell et al. (2014) [96]	Qatar	HCW	≥18	NR	10, 9, 1, 182
Goodwin et al. (2014) [97]	USA	Army recruits	17–36	1	1, 13, 5, 2062
Mathad et al. (2014) [98]	India	Pregnant women	≥18	NR	46, 12, 79, 206
Ribeiro-Rodrigues et al. (2014) [99]	Brazil	Contacts	0.5–87	77.3	159, 36, 14, 100
Sauzullo et al. (2014) [100]	Italy	HCW	25–60	3.1	34, 29, 0, 126
Song et al. (2014) [101]	Korea	Contacts	11–19	61	231, 430, 86, 2219
Adams et al. (2015) [102]	South Africa	HCW	≥18	92	293, 112, 24, 53
El-Sokkary et al. (2015) [103]	Egypt	HCW	≥18	92.4	26, 52, 12, 42
Gao et al. (2015) [104]	China	Mixed	≥5	50.6	2933, 2945, 1013, 13587
Goebel et al. (2015) [105]	Australia	Contacts	Any age	84	160, 194, 18, 91
He et al. (2015) [106]	Mongolia	HCW	19–77	36.4	122, 45, 276, 422
Howley et al. (2015) [107]	Vietnam, Philippines, Mexico	Migrants to USA	2–14	100	111, 553, 31, 1812
Jones-Lopez et al. (2015) [108]	Uganda	Contacts	≥10	2	182, 19, 15, 36
Lucet et al. (2015) [109]	France	HCW	≥18	97.4	95, 348, 18, 343
Ferrarini et al. (2016) [110]	Brazil	Contacts	≤15	98.3	31, 3, 3, 4
Al Hajoj et al. (2016) [111]	Saudi Arabia	HCW	≥18	90.6	227, 275, 172, 921
Biraro et al. (2016) [112]	Uganda	Contacts	0–30	78	62, 7, 92, 76
Bozkanat et al. (2016) [113]	Turkey	HCW	≥18	94.1	7, 21, 0, 6
Grare et al. (2010) [114]	France	Children	NR	41	5, 7, 0, 32
Lowenthal et al. (2016) [115]	USA	Immigrants	2–14	NR	142, 523, 3, 48
Marco Mourino et al. (2011) [116]	Spain	Prisoners	19–66	17	27, 13, 10, 99
Marquez et al. (2016) [117]	Uganda	Children	0–5	94	10, 114, 10, 343
Miramontes et al. (2015) [118]	USA	General	≥6	NR	127, 158, 176, 5603
Mostafavi et al. (2016) [119]	Iran	HCW	≥20	86	13, 26, 29, 176
Nienhaus et al. (2011) [120]	Germany, Portugal, France	HCW	≥18	NR	409, 654, 41, 523
Oren et al. (2016) [121]	USA	Migrant farmers	≥48	74	16, 8, 12, 32
Pavic et al. (2015) [122]	Croatia	Contacts	<5	98.8	18, 13, 8, 132
Reechaipichitkul et al. (2015) [123]	Thailand	Contacts	NR	86	15, 24, 5, 56
Rose et al. (2015) [124]	Canada	Contacts	0–17	42	27, 16, 4, 47
Salinas et al. (2015) [125]	Spain	Immigrants	12–18	26.75	140, 103, 2, 34
Sharma et al. (2017) [126]	India	Contacts	1–65	76	540, 187, 377, 394
Yoo et al. (2016) [127]	Korea	Contacts	NR	84	92, 71, 40, 241
Anibarro et al. (2011) [128]	Spain	Contacts	≥18	36	68, 14, 5, 50
Diel et al. (2008) [129]	Germany	Contacts	1–56	46	62, 181, 4, 354
Ferreira et al. (2015) [130]	Brazil	Contacts	≥18	86.7	19, 5, 9, 27
Nienhaus et al. (2008) [131]	Germany	HCW	18–67	37.5	15, 48, 10, 188
**Immune-competent,** **IGRA = T-SPOT.TB**					
Porsa et al. (2006) [132]	USA	Prisoners	≥18	NR	9, 28, 13, 359
Arend et al. (2007) [30]	Netherlands	Unvaccinated	≥17	0	103, 151, 39, 466
Rangaka et al. (2007) [133]	South Africa	Mixed	Any age	71	40, 21, 5, 8
Bienek & Chang (2009) [134]	USA	Unvaccinated	18–41	3	2, 0, 6, 318
Janssens et al. (2008) [135]	Switzerland	Contacts	16–83	80.6	78, 65, 37, 100
Leung et al. (2008) [136]	Hong Kong	Silicosis	≥18	1.5	72, 20, 14, 28
Soysal et al. (2008) [137]	Turkey	Healthy	Any age	83	7, 18, 0, 21
Girardi et al. (2009) [138]	Italy	HCW	≥18	37.4	37, 24, 5, 49
Hansted et al. (2009) [139]	Lithuania	Contacts	10–17	100	7, 20, 1, 17
		Low risk	10–17	100	3, 31, 2, 16
Kik et al. (2009) [41]	Netherlands	Contacts	≥16	NR	154, 85, 14, 29
Adetifa et al. (2010) [52]	Gambia	Contacts	0.5–14	59	43, 14, 27, 129
Leung et al. (2010) [140]	Hong Kong	Silicosis	≥18	3.5	168, 35, 36, 69
Borkowska et al. (2011) [141]	Poland	HCW	27–73	100	7, 4, 0, 6
Zhao et al. (2011) [142]	China	Students	17–24	0	11, 26, 16, 103
Larcher et al. (2012) [83]	Italy	HCW	19–64	38	24, 51, 35, 282
Nkurunungi et al. (2012) [143]	Uganda	Healthy	≤5	100	17, 6, 51, 218
Adams et al. (2015) [102]	South Africa	HCW	≥18	92	249, 126, 20, 55
Leung et al. (2015) [144]	Hong Kong	Contacts	5–64	66	254, 228, 89, 478
Spicer et al. (2015) [145]	USA	Mixed	0.3–16	72.5	5, 18, 0, 71
		Non-TB diseases	25–63	100	0, 3, 1, 26
**Immune-compromised,** **IGRA = QFT-GIT**					
Mendez-Echevarria et al. (2011) [146]	Spain	IMID	≥18	5.6	4, 3, 5, 37
Moon et al. (2011) [67]	Korea	Stem cell transplant	35–55	82	9, 24, 31, 146
Takahashi et al. (2007) [147]	USA	HIV	22–79	7.4	2, 5, 7, 259
Aichelburg et al. (2014) [148]	Austria	HIV	≥18	NR	24, 3, 13, 195
Balcells et al. (2008) [149]	Chile	HIV	21–71	88	9, 2, 8, 90
Bourgarit et al. (2015) [150]	France	HIV	≥18	60.6	20, 42, 14, 316
Casas et al. (2011) [151]	Spain	IMID	NR	26	43, 19, 13, 210
Casas et al. (2011) [152]	Spain	ESRD	NR	31.6	34, 10, 9, 42
Chkhartishvili et al. (2013) [153]	Georgia	HIV	≥18	94	25, 16, 44, 148
Gogus et al. (2010) [154]	Turkey	IMID	20–70	100	8, 17, 1, 12
Hanta et al. (2012) [155]	Turkey	IMID	≥18	92	24, 32, 10, 24
Hsia et al. (2012) [156]	Worldwide	IMID	All age	34.2	59, 150, 101, 1931
James et al. (2014) [157]	India	HIV	≥18	100	10, 16, 4, 18
Jones et al. (2007) [158]	USA	HIV	All age	2	5, 8, 6, 172
Karadag et al. (2010) [159]	Turkey	IMID	All age	100	19, 34, 2, 39
Khawcharoenporn et al. (2015) [160]	Thailand	HIV	17–65	73	8, 16, 12, 114
Kim et al. (2014) [161]	Korea	IMID	All age	70.7	56, 77, 12, 269
Kim et al. (2013) [162]	Korea	IMID	All age	NR	102, 133, 81, 408
Kim et al. (2015) [163]	Korea	IMID	All age	NR	52, 67, 26, 271
Latorre et al. (2014) [164]	Spain	IMID	≥18	NR	1, 6, 11, 81
Manuel et al. (2007) [165]	Canada	Liver transplant	≥18	82	18, 9, 16, 98
Matulis et al. (2008) [166]	Switzerland	IMID	≥18	83	10, 34, 5, 60
Minguez et al. (2012) [167]	Spain	IMID	≥18	5.6	4, 3, 5, 37
Moon et al. (2013) [168]	Korea	Stem cell transplant	35–55	82	9, 24, 31, 146
Papay et al. (2011) [169]	Austria	IMID	NR	100	6, 20, 9, 157
Ramos et al. (2013) [170]	Spain	IMID	16–82	19	13, 30, 2, 107
Ramos et al. (2012) [171]	Spain	HIV	15–85	15.8	21, 25, 8, 40
Sauzullo et al. (2010) [172]	Italy	IMID	18–80	8.7	27, 26, 5, 11
Talati et al. (2009) [173]	USA	HIV	22–79	7.4	2, 5, 7, 259
Vassilopoulos et al. (2011) [174]	Greece	IMID	≥18	76	17, 41, 15, 82
Hoffmann et al. (2010) [175]	Switzerland	Haemodialysis	30–87	18	5, 2, 4, 21
Mariette et al. (2012) [176]	France	IMID	All age	65.7	24, 114, 15, 239
Ponce de Leon et al. (2008) [177]	Peru	IMID	All age	80.2	21, 6, 24, 50
Scrivo et al. (2012) [178]	Italy	IMID	18–80	5.8	2, 11, 3, 82
Cho et al. (2016) [179]	Korea	IMID	NR	77.9	19, 16, 19, 148
Kurti et al. (2015) [180]	Hungary	IMID	18–30	100	7, 28, 5, 126
Kussen et al. (2016) [181]	Brazil	HIV	≥18	78	9, 4, 12, 115
Palomar et al. (2011) [182]	Spain	Haemodialysis	NR	42.6	7, 9, 3, 26

* TST+/IGRA+, TST+/IGRA-, TST-/IGRA+, TST-/IGRA-.

ESRD, end stage renal disease; IGRA, interferon gamma release assay; IMID, immune-mediated inflammatory disease; HCW, healthcare worker; NR, not reported; QFT-GIT, QuantiFERON-TB Gold In Tube; TB, tuberculosis.

**Fig 2:**
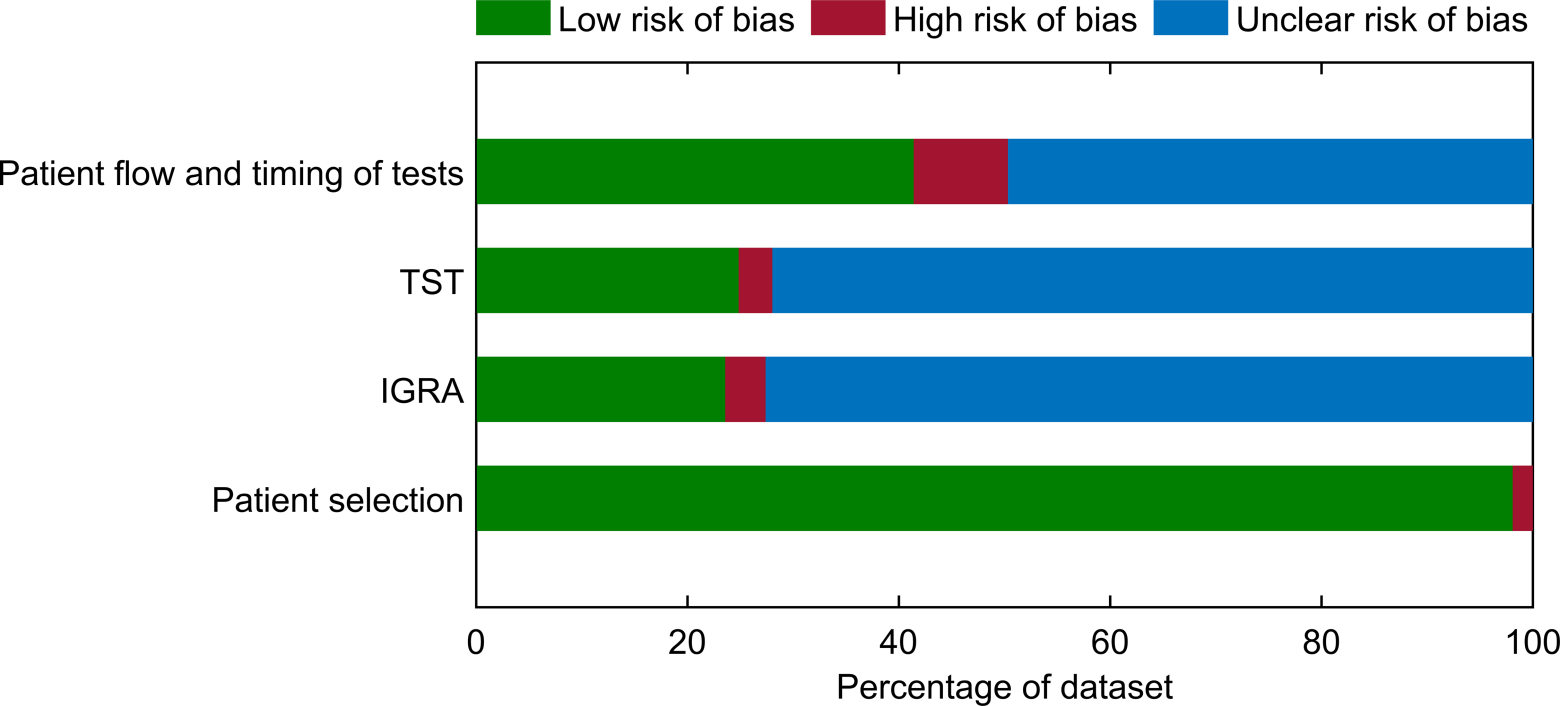
Summary of quality assessment results. Risk of Bias of each QUADAS-2 domain presented as percentages across the 157 included studies. IGRA; interferon-gamma release assay; TST, tuberculin skin test.

The results of the quality assessment of the included studies are summarised in Fig 2 and presented for each individual study in S1 Table. Many studies did not report all the information that could be used to fully assess the quality of the study. For the “patient selection” domain, most studies (154/157, 98%) were deemed to have low risk of bias (Fig 2). The remaining 2% were considered to have high risk of bias because these studies used a case-control study design in which the status of LTBI were known prior to the test. For the “diagnostic test domains”, risk of bias could not be assessed for the majority of studies because it was unknown whether the results of one test were interpreted without knowledge of the results of the other test (Fig 2). Nine percent (14/157) of the studies were deemed to have high risk of bias for the “patient flow and timing of tests domain” because there were participants excluded from the analysis without explanation given (Fig 2). There was unclear risk of bias for this domain for 50% (78/157) of the studies because the interval between the two tests was not reported (Fig 2).

Table 2 shows the estimated sensitivity and specificity of TST, QFT-GIT and T-SPOT.TB in different populations. In immune-competent non-BCG-vaccinated adults, TST has better sensitivity (84% versus 52%) and slightly better specificity (100% versus 97%) than QFT-GIT. BCG vaccination significantly reduces the specificity of TST, from 100% in non-vaccinated subjects to 79% in BCG-vaccinated subjects; whereas the effect of BCG on the specificity of QFT-GIT is modest (Table 2). T-SPOT.TB has comparable specificity (97% for both tests) and better sensitivity (68% versus 52%) than QFT-GIT in immune-competent adults. In immune-compromised adults, QFT-GIT is less sensitive than TST (46% versus 71%) whereas the specificity of both tests is comparable (97% versus 99% in non-BCG-vaccinated adults, 93% for both tests in BCG-vaccinated adults) (Table 2). QFT-GIT and TST have comparable specificity in non-BCG-vaccinated children; however the former is less sensitive than the latter (Table 2). The specificity of QFT-GIT in BCG-vaccinated children is not affected by BCG and is substantially better than that of TST (98% versus 82%) (Table 2).

**Table 2:** Estimated sensitivity and specificity of TST and IGRAs in different population groups.

Parameter	Diagnostic test	Immune-competent adults* median (95% CrI)	Immune-compromised adults† median (95% CrI)	Immune-competent children* median (95% CrI)
Sensitivity (%)	QFT-GIT	52 (50–53)	46 (43–49)	73 (70–76)
	TST	84 (82–85)	71 (66–75)	82 (79–84)
Specificity (%)	QFT-GIT (non-BCG)	97 (96–97)	97 (96–98)	98 (97–99)
	QFT-GIT (BCG)	93 (92–94)	93 (92–95)	98 (97–99)
	TST (non-BCG)	100 (99–100)	99 (97–100)	98 (96–99)
	TST (BCG)	79 (76–82)	93 (91–96)	82 (81–83)

*TST cut-off value = 10 mm

†TST cut-off value = 5 mm

BCG, Bacillus Calmette-Guérin; CrI, credible interval; QFT-GIT, QuantiFERON-TB Gold In Tube; TST, tuberculin skin test.

**Fig 3:**
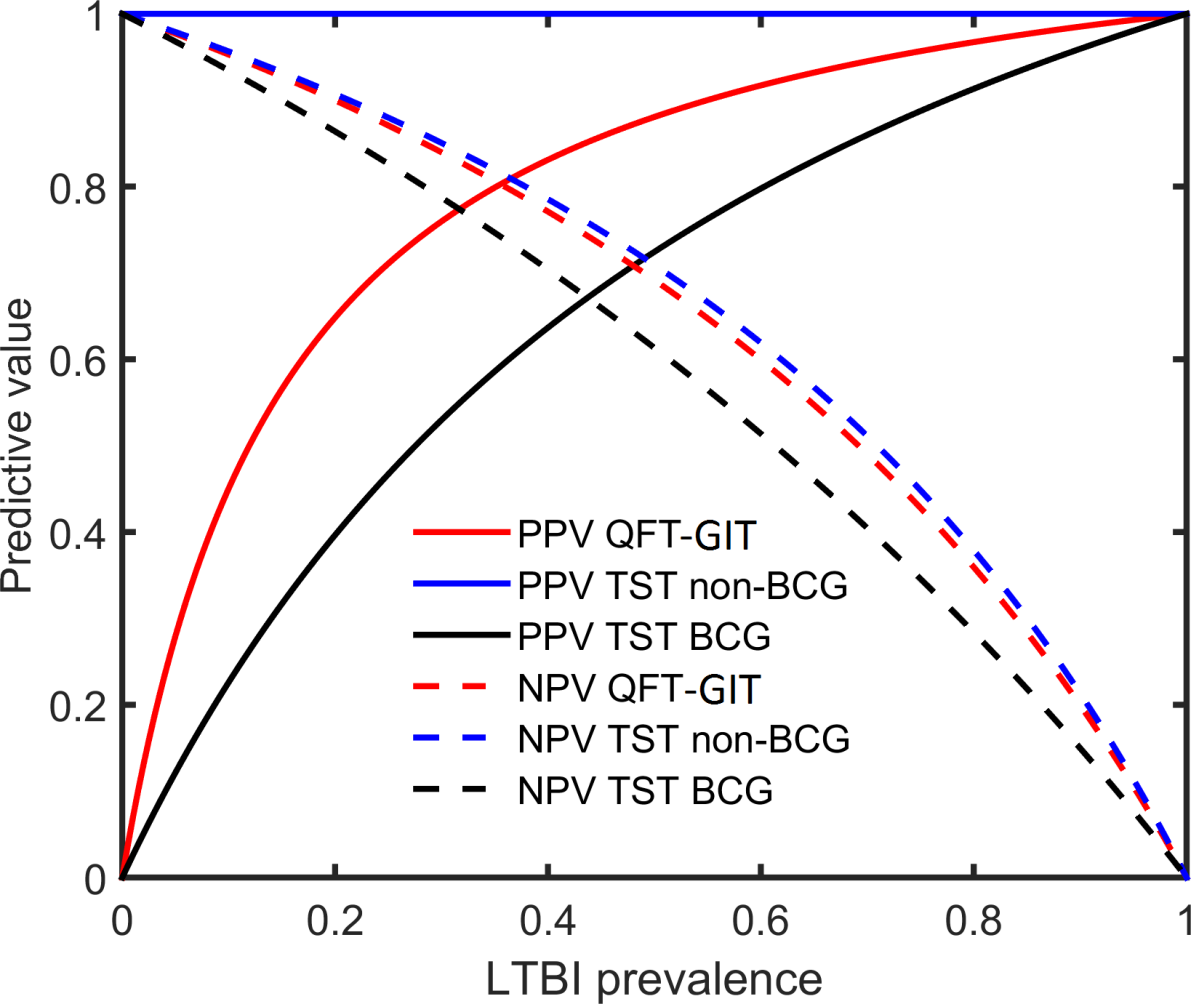
Relationship between prevalence and predictive value in immune-competent adults. BCG, Bacillus Calmette-Guérin; LTBI, latent tuberculosis infection; NPV, negative predictive value; PPV, positive predictive value; QFT-GIT, QuantiFERON-TB Gold In Tube; TB, tuberculosis; TST, tuberculin skin test.

The mean prevalence of LTBI among the populations where the studies were performed was estimated to be 49% (standard deviation ± 27%). The relationship between prevalence and predictive values is shown in Fig 3. In a high-prevalence setting (prevalence > 50%), QFT-GIT has a PPV of at least 88% and a NPV value of at most 69%. The PPV of TST is around 100% in non-BCG-vaccinated and at least 73% in BCG-vaccinated subjects. The NPV of TST was estimated to be 71% and 61% in these populations, respectively.

## Discussion

Accurate identification and subsequent treatment of LTBI is essential to TB control and elimination. The lack of a gold standard for diagnosing LTBI means that the true prevalence of the disease is unknown, and the estimations of the sensitivity and specificity of diagnostic tests are unreliable. This study represents the most comprehensive Bayesian latent class analysis of published data on the performance of TST and IGRAs for the diagnosis of LTBI. We have confirmed that IGRAs have high specificity but that these tests have considerably lower sensitivity than TST in immune-competent populations than had previously been demonstrated [6,7,183]. A meta-analysis by Pai et al.[*7*] estimated the pooled sensitivity of QFT and TST to be 70% and 77%, respectively; the specificity of QFT to be 96–99%; and the specificity of TST in non-BCG-vaccinated and BCG-vaccinated populations to be 97% and 59%, respectively. Our estimate of the sensitivity of QFT-GIT is lower than that of Pai et al. [7]; however it should be noted that the sensitivity of QFT in Pai et al. [7] was estimated in patients with active TB as a surrogate for LTBI. It is plausible that the cellular immune response, which is the measure of QFT, is different between LTBI and active TB disease, being higher with the latter [5]. Using a similar latent class modelling approach, Sadatsafavi et al. [6] estimated the sensitivity and specificity of QFT in immune-competent adults to be 64.2% and 99.6%, respectively. However, methodological differences make comparison between our results and those of Sadatsafavi et al. [6] challenging. Sadatsafavi et al. [6], conducted in 2008, is nearly a decade old and only included a very limited number (nineteen) of studies. Since then, a great amount of new studies that compared the diagnostic performance of IGRAs and TST in this setting have been published. Indeed, our search has found that since the study of Sadatsafavi et al. [6] was conducted, there have been 132 new studies that are included in our analysis. Sadatsafavi et al. [6] combined all versions of QFT in their analysis, assuming no difference between these tests; whereas our study included only the latest QFT-GIT version, which replaced the discontinued older QFT versions. In addition, Sadatsafavi et al. [6] only included immune-competent adults; whereas we included not only immune-competent adults but also children and immune-compromised individuals. The study of Sadatsafavi et al. [6] is limited to a single database and to studies in English language only. Single database and English-only language restrictions are likely to result in an incomplete coverage of the literature and biased estimates.

Conventional meta-analysis of diagnostic tests simply entails pooling of data to provide pooled estimates of test sensitivity and specificity. Simple pooling of data may cause serious bias due to confounding of disease prevalence in the contributing studies [184]. Our latent class modelling approach accounts for the imperfect nature of the tests; and allows us to estimate not only diagnostic parameters (i.e. sensitivity, specificity, predictive values), but also disease prevalence. Unlike conventional meta-analysis, Bayesian latent class modelling incorporates prior information on sensitivity, specificity and disease prevalence, improving the precision of model estimates for these parameters. It also allows for the quantification of the effect of BCG on the performance of the tests, which otherwise is impossible to measure in conventional epidemiological studies and meta-analysis. Before our study, there had been no formal quantification of the effect of BCG on the specificity of IGRAs; even though it is generally thought that such effect, if any, is modest based on the biological mechanism of the tests, rather than on empirical data [185]. Our study is the first to quantify the effect of BCG on the specificity of IGRAs. We have found that such effect is minimal, confirming this hypothesis. We have also been able to quantify the decrement in specificity of TST in BCG-vaccinated subjects. To date, studies that investigated the impact of BCG on TST have only reported such effect as relative risk or odds ratio of having positive TST results between subjects with and without BCG [22,23,167]. We have found that BCG negatively affects the performance of TST, reducing the specificity of the test by 21% in the general population. In contrast, QFT-GIT has reasonable sensitivity and superior specificity in BCG-vaccinated subjects, supporting the recommendation that QFT-GIT should be the preferred diagnostic test of LTBI in this setting [177,178]. Of note, the effect of BCG on the specificity of the tests was inferred in our model based on the rates of BCG vaccination. We did not take into account other factors that are known to potentially affect the diagnostic performance of TST including age at vaccination and time since vaccination because of the lack of data [179]. An important assumption underlying Bayesian latent class models is the assumption of conditional independence between the two test observations [25]. Using the LORC method, we estimated the z-score to be 0.8, falling within the ± 1.96 range, indicating no violation of the conditional independence assumption. To explore the potential effects that studies deemed to be of high risk of bias may have on the results, we performed an analysis in which these studies were excluded. We found that our results were robust to the inclusion (or exclusion) of these studies (S2 Table).

Immune-compromised patients have an increased risk of LTBI reactivation [5]. Screening for LTBI is therefore required prior to commencement of immunosuppressive therapies [5]. To date, data on the performance of diagnostic tests for LTBI in immune-compromised subjects are limited and the few published studies evaluating the performance of TST and QFT-GIT show conflicting results [5,186]. We have found that both tests are specific but have suboptimal sensitivity in immune-compromised patients. We believe that more data on the performance of TST and QFT-GIT in this population group are required.

The limitations of our study must be considered. Our results are derived from studies where the estimates of LTBI prevalence vary widely. This is due to the heterogeneity in study settings, populations and methodology of the included studies. Bayesian analysis requires prior information on model parameters. One criticism of Bayesian latent class models is that they may be sensitive to the choice of prior information. This may particularly be the case when there are limited observed data. When the number of observed data are large, as in our study, these begin to dominate any prior information. We believe that we have used the most informative priors obtained from the literature. Furthermore, we performed sensitivity analysis and found that our results are not sensitive to choice of prior (S3 Table).

In conclusion, our study represents the most comprehensive Bayesian latent class analysis of the diagnostic accuracy of TST and IGRAs derived from all published agreement data. Our results challenge the current beliefs about the performance of LTBI screening tests and provide important information to guide choice of tests for LTBI screening that will enhance the millennium goals for elimination of TB. Our findings show that IGRAs may be inferior to TST for diagnosing LBTI in non-BCG-vaccinated populations. For BCG-vaccinated individuals, IGRAs appear to be a more favourable choice. IGRAs will therefore allow physicians and TB controllers to better understand the background prevalence of LTBI for targeted preventive therapy in settings where BCG vaccination is widely administered. QFT-GIT and TST have suboptimal sensitivity in immune-compromised patients and results should be interpreted with caution. A combination of both tests could potentially overcome the problems of false-positives in this setting. Considerations regarding cost-effectiveness, logistics, availability for clinicians and patient acceptability should be taken into account to decide which test to use for the diagnosis of LTBI.

## Supporting information

S1 Text PubMed search strategy. (PDF)

S2 Text Description of the QUADAS-2 critical appraisal checklist. (PDF)

S3 Text Formulae for positive predictive value (PPV) and negative predictive value (NPV). (PDF)

S1 Table Results of quality assessment using the QUADAS-2 checklist. IGRA, interferon gamma release assay; N, No; Q, Question; TST, tuberculin skin test; U, Unclear; Y, Yes.
(PDF)

S2 Table Sensitivity of results to exclusion of studies deemed to be of high risk of bias. *Results are for immune-competent adults. BCG, Bacillus Calmette-Guérin; CrI, credible interval; QFT-GIT, QuantiFERON-TB Gold In Tube; TB, tuberculosis; TST, tuberculin skin test.
(PDF)

S3 Table Sensitivity of results to prior distributions. *Results are for immune-competent adults. BCG, Bacillus Calmette-Guérin; CrI, credible interval; QFT-GIT, QuantiFERON-TB Gold In Tube; TB, tuberculosis; TST, tuberculin skin test.
(PDF)

S4 Table PRISMA checklist. (PDF)
