# Supplementary material for: Interferon-gamma release assay for the diagnosis of latent tuberculosis infection: A latent-class analysis
Source: PLoS One. 2017 Nov 28;12(11):e0188631. doi: 10.1371/journal.pone.0188631 (PMC5705142; doi:10.1371/journal.pone.0188631)
Supplement: S1 Text — (PDF) [file pone.0188631.s001.pdf]

PubMed search strategy

Search algorithm:

(tuberculin[mesh]) OR “TST” OR “Mantoux”) and (“interferon gamma release assay” OR “interferon gamma assay” OR “QuantiFero*” OR “IGRA” OR “T-SPO*” OR “TSPO*” OR “Elispot” OR CFP10 OR ESAT6) and (tuberculosis[mesh]).

Restrictions: Humans
