# Supplementary material for: Interferon-gamma release assay for the diagnosis of latent tuberculosis infection: A latent-class analysis
Source: PLoS One. 2017 Nov 28;12(11):e0188631. doi: 10.1371/journal.pone.0188631 (PMC5705142; doi:10.1371/journal.pone.0188631)
Supplement: S2 Text — (PDF) [file pone.0188631.s002.pdf]

Description of the QUADAS-2 critical appraisal checklist

Domain 1. Patient selection

Risk of bias: Could the selection of patients have introduced bias?

- Signalling question 1: Was a consecutive or random sample of patients enrolled?

  We scored "Yes" if a consecutive or random sample of eligible patients was enrolled; "No" if patients were selected by convenience; and "Unclear" if the study did not report the manner in which patients were enrolled.
- Signalling question 2: Was a case-control design avoided?

  We scored "Yes" if a case-control design was avoided; "No" if the study employed a case-control design; and "Unclear" if study design was not reported.
- Signalling question 3: Did the study avoid inappropriate exclusions?

  We scored "Yes" if no inappropriate exclusion was noted; "No" if inappropriate exclusions were noted such as patients were excluded because of prior knowledge about them other than their tuberculosis infection/disease status, or their test results were not available because the technician forgot to record them.

We considered Risk of Bias to be "Low Risk" if we scored "Yes" for all of the three signalling questions; "High Risk" if we scored "No" for either signalling question 1 or signalling question 2; and "Unclear" if we were unclear about study selection or case-control study design.

Concerns regarding applicability: Is there concern that the included patients do not match the review question?

Our study aimed to investigate the diagnostic performance of tuberculin skin test (TST) and interferon gamma release assays (IGRAs) for the diagnosis of latent tuberculosis infection (LTBI). We judged "Low Applicability Concern" if we scored "Yes" for all of the three signalling questions; "High Applicability Concern" if we scored "No" for either signalling question 1 or signalling question 2; and "Unclear Applicability Concern" if we could not tell.

Domain 2. Interferon gamma release assay (IGRA) test

Risk of bias: Could the conduct or interpretation of the test have introduced bias?

- Signalling question 1: Were the IGRA test results interpreted without knowledge of the results of TST test?

  We scored "Yes" if the IGRA test results were interpreted without knowledge of the results of TST test; "No" if blinding to test results were not done; and "Unclear" if this was not stated.

- Signalling question 2: If a threshold was used, was it pre-specified?

  We scored "Yes" if an IGRA cut-off value was pre-specified or it was stated that "the cut-off value as per the manufacturer's guidelines was used"; and "No" if this was not stated.

We considered Risk of Bias to be "Low Risk" if we scored "Yes" for both signalling questions; "High Risk" if we scored "No" for either of the questions; and "Unclear" if we were unclear about blinding status or cut-off value.

Concerns regarding applicability: Is there concern that IGRA test, its conduct, or interpretation differ from the review question?

We judged "Low Applicability Concern" if interpretation of test results was blinded, threshold value was specified, and the test was performed as per the manufacturer's guidelines. We judged "High Applicability Concern" if blinding in test result interpretation was not done, threshold value was not specified or the test was not carried out as per the manufacturer's recommendations. We judged "Unclear Applicability Concern" if the blinding status of the study and threshold value were unclear.

Domain 3. Tuberculin skin test (TST)

Risk of bias: Could the conduct or interpretation of the test have introduced bias?

Although TST has been traditionally used for the diagnosis of LTBI because of its low cost, it is not considered as a gold standard for testing LTBI. Indeed, there is currently a lack of a reference test for diagnosing LTBI. Therefore, the question "Is the reference standard likely

to correctly classify the target condition?" in QUADAS-2 is not applicable. Instead, we used the same signalling questions for TST as in the case of IGRA test (Domain 2).

- Signalling question 1: Were TST test results interpreted without knowledge of the results of IGRA test?

  We scored "Yes" if the TST test results were interpreted without knowledge of the results of IGRA test; "No" if blinding to test results were not done; and "Unclear" if this was not stated.

- Signalling question 2: If a threshold was used, was it pre-specified?

  We scored "Yes" if a TST cut-off value was pre-specified; and "No" if this was not stated.

We considered Risk of Bias to be "Low Risk" if we scored "Yes" for both signalling questions; "High Risk" if we scored "No" for either of the questions; and "Unclear" if we were unclear about blinding status or cut-off value.

Concerns regarding applicability: Is there concern that TST test, its conduct, or interpretation differ from the review question?

We judged "Low Applicability Concern" if interpretation of test results was blinded and TST cut-off value was specified. We judged "High Applicability Concern" if blinding in test result interpretation was not done or TST cut-off value was not specified. We judged "Unclear Applicability Concern" if the blinding status of the study and cut-off value were unclear.

Domain 4. Flow and timing

Risk of bias: Could the patient flow have introduced bias?

- Signalling question 1: Was there an appropriate interval between IGRA and TST tests?

  We scored "Yes" if the two tests were paired or performed within 48 hours from one another.

- Signalling question 2: Were all patients included in the analysis?

  We answered this question by comparing the number of participants included in the study and the number of individuals included in the 2x2 tables or test agreement data. We scored 'Yes' if the number of participants enrolled was

stated and corresponded to the number included in the analysis or if exclusions were adequately described. We scored 'No' if there were participants missing or excluded from the analysis and there was no explanation given. We scored "Unclear" if we could not tell, e.g. because the number of participants enrolled and/or number of participants included in the analysis was not clearly stated.

We considered Risk of Bias to be "Low Risk" if we scored "Yes" for both signalling questions; "High Risk" if we scored "No" for either of the questions; and "Unclear" if we were unclear about the flow or timing of study.
