# Supplementary material for: Interferon-gamma release assay for the diagnosis of latent tuberculosis infection: A latent-class analysis
Source: PLoS One. 2017 Nov 28;12(11):e0188631. doi: 10.1371/journal.pone.0188631 (PMC5705142; doi:10.1371/journal.pone.0188631)
Supplement: S3 Text — (PDF) [file pone.0188631.s003.pdf]

Formulae for positive predictive value (PPV) and negative predictive value (NPV)

$$PPV = P(D + |T +) = \frac{P(T + |D +)P(D+)}{P(T+)} = \frac{P(T + |D +)P(D+)}{P(T + |D +)P(D +) + P(T + |D -)P(D-)}$$

$$= \frac{S\pi}{S\pi + (1 - C)(1 - \pi)};$$

$$NPV = P(D - |T -) = \frac{P(T - |D -)P(D-)}{P(T-)} = \frac{P(T - |D -)P(D-)}{P(T - |D +)P(D +) + P(T - |D -)P(D-)}$$

$$= \frac{C(1 - \pi)}{(1 - S)\pi + C(1 - \pi)}.$$

where *D* is disease status; *T* is diagnostic test results; π is prevalence of the disease in the population; *S* is sensitivity of the diagnostic test being considered; and *C* is specificity of the diagnostic test being considered.
