# Supplementary material for: Interferon-gamma release assay for the diagnosis of latent tuberculosis infection: A latent-class analysis
Source: PLoS One. 2017 Nov 28;12(11):e0188631. doi: 10.1371/journal.pone.0188631 (PMC5705142; doi:10.1371/journal.pone.0188631)
Supplement: S1 Table — IGRA, interferon gamma release assay; N, No; Q, Question; TST, tuberculin skin test; U, Unclear; Y, Yes. (PDF) [file pone.0188631.s004.pdf]

S1 Table. Results of quality assessment using the QUADAS-2 checklist.

Q = Question. Y = Yes. N = No. U = Unclear. IGRA = interferon gamma release assay. TST = tuberculin skin test.

	Patient selection					IGRA test				TST				Flow and timing		
Reference	Q1	Q2	Q3	Risk of bias	Applicability concern	Q1	Q2	Risk of bias	Applicability concern	Q1	Q2	Risk of bias	Applicability concern	Q1	Q2	Risk of bias
Diel et al. (2006)[1]	Y	Y	Y	L	L	Y	Y	L	L	Y	Y	L	L	Y	Y	L
Nakaoka et al. (2006)[2]	Y	Y	Y	L	L	Y	Y	L	L	Y	Y	L	L	U	Y	U
Porsa et al. (2006)[3]	Y	Y	Y	L	L	U	Y	U	U	U	Y	U	U	U	Y	U
Tsiouris et al. (2006)[4]	Y	Y	Y	L	L	U	Y	U	U	U	Y	U	U	U	N	H
Adetifa et al. (2007)[5]	Y	Y	Y	L	L	U	Y	U	U	U	Y	U	U	N	Y	H
Arend et al. (2007)[6]	Y	N	Y	H	H	U	N	H	U	U	Y	U	U	U	Y	U
Dogra et al. (2007)[7]	Y	Y	Y	L	L	Y	Y	L	L	Y	Y	L	L	U	Y	U
Franken et al. (2007)[8]	Y	Y	Y	L	L	U	Y	U	U	U	Y	U	U	U	Y	U
Rangaka et al. (2007)[9]	Y	Y	Y	L	L	Y	N	H	H	Y	Y	L	L	U	Y	U
Silverman et al. (2007)[10]	Y	Y	Y	L	L	U	Y	U	U	U	Y	U	U	U	Y	U
Chun et al. (2008)[11]	Y	Y	Y	L	L	U	N	H	H	U	Y	U	U	U	Y	U
Bienek and Chang (2009)[12]	Y	Y	Y	L	L	U	Y	U	U	U	Y	U	U	U	Y	U
Janssens et al. (2008)[13]	Y	Y	Y	L	L	U	Y	U	U	U	Y	U	U	Y	Y	L
Leung et al. (2008)[14]	Y	Y	Y	L	L	U	N	H	H	U	Y	U	U	U	Y	U
Mirtskhulava et al. (2008)[15]	Y	Y	Y	L	L	U	Y	U	U	U	Y	U	U	Y	Y	L
Petrucci et al. (2008)[16]	Y	Y	Y	L	L	U	Y	U	U	U	Y	U	U	U	Y	U

Soysal et al. (2008)[17]	Y	Y	Y	L	L	U	Y	U	U	U	Y	U	U	U	Y	U
Baker et al. (2009)[18]	Y	Y	Y	L	L	Y	Y	L	L	Y	Y	L	L	Y	Y	L
Bianchi et al. (2009)[19]	Y	Y	Y	L	L	Y	Y	L	L	Y	Y	L	L	Y	Y	L
Fox et al. (2009)[20]	Y	Y	Y	L	L	Y	Y	L	L	Y	Y	L	L	Y	Y	L
Girardi et al. (2009)[21]	Y	Y	Y	L	L	U	Y	U	U	U	Y	U	U	N	Y	H
Hansted et al. (2009)[22]	Y	N	Y	H	H	U	Y	U	U	U	Y	U	U	Y	Y	L
Herrmann et al. (2009)[23]	Y	Y	Y	L	L	U	Y	U	U	Y	Y	L	L	U	Y	U
Kik et al. (2009)[24]	Y	Y	Y	L	L	U	Y	U	U	U	N	H	H	U	Y	U
Kim et al. (2009)[25]	Y	Y	Y	L	L	U	Y	U	U	U	Y	U	U	U	Y	U
Lien et al. (2009)[26]	Y	Y	Y	L	L	U	Y	U	U	U	Y	U	U	U	Y	U
Lighter et al. (2009)[27]	Y	Y	Y	L	L	U	Y	U	U	U	Y	U	U	U	Y	U
Machado et al. (2009)[28]	Y	Y	Y	L	L	U	Y	U	U	U	Y	U	U	U	Y	U
Ringshausen et al. (2009)[29]	Y	Y	Y	L	L	Y	Y	L	L	Y	Y	L	L	U	Y	U
Saracino et al. (2009)[30]	Y	Y	Y	L	L	U	Y	U	U	U	Y	U	U	U	Y	U
Torres Costa et al. (2009)[31]	Y	Y	Y	L	L	U	Y	U	U	U	Y	U	U	Y	N	H
Tripodi et al. (2009)[32]	Y	Y	Y	L	L	U	Y	U	U	U	Y	U	U	Y	Y	L
Vinton et al. (2009)[33]	Y	Y	Y	L	L	U	Y	U	U	Y	Y	L	L	U	Y	U
Zhao et al. (2009)[34]	Y	Y	Y	L	L	Y	Y	L	L	Y	N	H	H	U	Y	U
Adetifa et al. (2010)[35]	Y	Y	Y	L	L	U	Y	U	U	U	Y	U	U	Y	Y	L
Costa et al. (2010)[36]	Y	Y	Y	L	L	Y	Y	L	L	Y	Y	L	L	Y	Y	L
Grare et al. (2010)[37]	Y	Y	Y	L	L	U	Y	U	U	U	Y	U	U	Y	Y	L
Huang et al. (2010)[38]	Y	Y	Y	L	L	U	Y	U	U	U	Y	U	U	Y	Y	L

Jong Lee et al. (2010)[39]	Y	Y	Y	L	L	U	Y	U	U	U	Y	U	U	U	Y	U
Katsenos et al. (2010)[40]	Y	Y	Y	L	L	U	Y	U	U	U	Y	U	U	N	Y	H
Lee et al. (2010)[41]	Y	Y	Y	L	L	U	Y	U	U	U	Y	U	U	U	Y	U
Leung et al. (2010)[42]	Y	Y	Y	L	L	U	Y	U	U	U	Y	U	U	U	Y	U
Torres Costa et al. (2010)[43]	Y	Y	Y	L	L	U	Y	U	U	U	Y	U	U	N	Y	H
Thomas et al. (2010)[44]	Y	Y	Y	L	L	U	Y	U	U	U	Y	U	U	U	N	H
Tsolia et al. (2010)[45]	Y	Y	Y	L	L	U	Y	U	U	U	Y	U	U	U	Y	U
Caglayan et al. (2011)[46]	Y	Y	Y	L	L	U	U	U	U	U	Y	U	U	U	Y	U
Diel et al. (2011)[47]	Y	Y	Y	L	L	Y	Y	L	L	Y	Y	L	L	Y	Y	L
Kasambira et al. (2011)[48]	Y	Y	Y	L	L	U	Y	U	U	U	Y	U	U	Y	N	H
Kus et al. (2011)[49]	Y	Y	Y	L	L	U	Y	U	U	U	Y	U	U	U	Y	U
Legesse et al. (2011)[50]	Y	Y	Y	L	L	U	Y	U	U	U	Y	U	U	Y	Y	L
Moon et al. (2011)[51]	Y	Y	Y	L	L	Y	Y	L	L	Y	Y	L	L	U	Y	U
Moyo et al. (2011)[52]	Y	Y	Y	L	L	U	Y	U	U	U	Y	U	U	U	Y	U
Pavic et al. (2011)[53]	Y	Y	Y	L	L	U	Y	U	U	U	Y	U	U	Y	Y	L
Rafiza et al. (2011)[54]	Y	Y	Y	L	L	U	Y	U	U	U	Y	U	U	U	Y	U
Shanaube et al. (2011)[55]	Y	Y	Y	L	L	U	Y	U	U	U	N	H	H	U	Y	U
Talebi-Taher et al. (2011)[56]	Y	Y	Y	L	L	U	Y	U	U	U	Y	U	U	U	Y	U
Torres Costa et al. (2011)[57]	Y	Y	Y	L	L	Y	Y	L	L	Y	Y	L	L	Y	Y	L
Torres Costa et al. (2011)[58]	Y	Y	Y	L	L	U	Y	U	U	U	Y	U	U	Y	Y	L

Weinfurter et al. (2011)[59]	Y	Y	Y	L	L	U	Y	U	U	U	Y	U	U	Y	N	H
Yassin et al. (2011)[60]	Y	Y	Y	L	L	U	Y	U	U	U	Y	U	U	U	Y	U
Zhao et al. (2011)[61]	Y	Y	Y	L	L	U	Y	U	U	U	Y	U	U	U	N	H
Bergot et al. (2012)[62]	Y	Y	Y	L	L	U	Y	U	U	U	Y	U	U	U	Y	U
Di Renzi et al. (2012)[63]	Y	Y	Y	L	L	U	N	H	H	U	Y	U	U	U	Y	U
He et al. (2012)[64]	Y	Y	Y	L	L	U	Y	U	U	U	Y	U	U	U	Y	U
Jeong et al. (2012)[65]	Y	Y	Y	L	L	U	Y	U	U	U	Y	U	U	Y	Y	L
Jo et al. (2012)[66]	Y	Y	Y	L	L	U	Y	U	U	U	Y	U	U	Y	Y	L
Jung da et al. (2012)[67]	Y	Y	Y	L	L	U	Y	U	U	U	Y	U	U	Y	Y	L
Larcher et al. (2012)[68]	Y	Y	Y	L	L	U	Y	U	U	U	Y	U	U	U	Y	U
Nkurunungi et al. (2012)[69]	Y	Y	Y	L	L	Y	Y	L	L	Y	Y	L	L	Y	Y	L
Onur et al. (2012)[70]	Y	Y	Y	L	L	U	Y	U	U	U	Y	U	U	Y	Y	L
Pattnaik et al. (2012)[71]	Y	Y	Y	L	L	U	Y	U	U	U	Y	U	U	Y	Y	L
Zwerling et al. (2012)[72]	Y	Y	Y	L	L	U	Y	U	U	U	Y	U	U	U	N	H
Jo et al. (2013)[73]	Y	Y	Y	L	L	U	Y	U	U	U	Y	U	U	U	Y	U
Serrano-Escobedo et al. (2013)[74]	Y	Y	Y	L	L	U	Y	U	U	U	Y	U	U	U	Y	U
Whitaker et al. (2013)[75]	Y	Y	Y	L	L	U	Y	U	U	U	Y	U	U	U	Y	U
Zwerling et al. (2013)[76]	Y	Y	Y	L	L	U	Y	U	U	U	Y	U	U	U	Y	U
Aichelburg et al. (2014)[77]	Y	Y	Y	L	L	U	Y	U	U	U	Y	U	U	Y	Y	L
Alvarez et al. (2014)[78]	Y	Y	N	L	L	Y	Y	L	L	Y	Y	L	L	N	Y	H

Charisis et al. (2014)[79]	Y	Y	Y	L	L	U	Y	U	U	U	Y	U	U	Y	Y	L
de Souza et al. (2014)[80]	Y	Y	Y	L	L	U	Y	U	U	U	Y	U	U	Y	Y	L
Erkens et al. (2014)[81]	Y	Y	Y	L	L	U	Y	U	U	U	Y	U	U	U	Y	U
Garazzino et al. (2014)[82]	Y	Y	Y	L	L	U	Y	U	U	U	N	H	H	U	Y	U
Garcell et al. (2014)[83]	Y	Y	Y	L	L	N	Y	H	H	N	N	H	H	U	Y	U
Goodwin et al. (2014)[84]	Y	Y	Y	L	L	U	Y	U	U	U	Y	U	U	U	Y	L
Mathad et al. (2014)[85]	Y	Y	Y	L	L	U	Y	U	U	U	Y	U	U	Y	Y	L
Ribeiro-Rodrigues et al. (2014)[86]	Y	Y	Y	L	L	Y	Y	L	L	Y	Y	L	L	U	Y	U
Sauzullo et al. (2014)[87]	Y	Y	Y	L	L	U	Y	U	U	U	Y	U	U	U	Y	U
Song et al. (2014)[88]	Y	Y	Y	L	L	U	Y	U	U	U	Y	U	U	Y	Y	L
Adams et al. (2015)[89]	Y	Y	Y	L	L	U	Y	U	U	U	Y	U	U	N	N	H
El-Sokkary et al. (2015)[90]	Y	Y	Y	L	L	U	Y	U	U	U	Y	U	U	U	Y	U
Gao et al. (2015)[91]	Y	Y	Y	L	L	U	Y	U	U	U	Y	U	U	Y	Y	L
Goebel et al. (2015)[92]	Y	Y	Y	L	L	U	Y	U	U	U	Y	U	U	U	Y	U
He et al. (2015)[93]	Y	Y	Y	L	L	U	Y	U	U	U	Y	U	U	U	Y	U
Howley et al. (2015)[94]	Y	Y	Y	L	L	U	Y	U	U	U	Y	U	U	Y	Y	L
Jones-Lopez et al. (2015)[95]	Y	Y	Y	L	L	U	Y	U	U	U	Y	U	U	Y	Y	L
Leung et al. (2015)[96]	Y	Y	Y	L	L	U	Y	U	U	U	Y	U	U	Y	Y	L
Spicer et al. (2015)[97]	Y	Y	Y	L	L	U	Y	U	U	U	Y	U	U	Y	Y	L
Lucet et al. (2015)[98]	Y	Y	Y	L	L	U	Y	U	U	U	Y	U	U	U	Y	U

Ferrarini et al. (2016)[99]	Y	N	Y	H	H	U	Y	U	U	U	Y	U	U	U	N	H
Al Hajoj et al. (2016)[100]	Y	Y	Y	L	L	U	Y	U	U	U	Y	U	U	U	Y	U
Biraro et al. (2016)[101]	Y	Y	Y	L	L	U	Y	U	U	U	Y	U	U	U	Y	U
Bozkanat et al. (2016)[102]	Y	Y	Y	L	L	U	Y	U	U	U	Y	U	U	Y	Y	L
Grare et al. (2010)[103]	Y	Y	U	L	L	U	Y	U	U	U	Y	U	U	U	U	U
Lowenthal et al. (2016)[104]	Y	Y	Y	L	L	U	U	U	U	U	Y	U	U	U	U	U
Marco Mourino et al. (2011) [105]	Y	Y	Y	L	L	U	Y	U	U	U	Y	U	U	Y	Y	L
Marquez et al. (2016)[106]	Y	Y	Y	L	L	Y	Y	L	L	Y	Y	L	L	Y	Y	L
Miramontes et al. (2015)[107]	Y	Y	Y	L	L	U	Y	U	U	U	Y	U	U	Y	Y	L
Mostafavi et al. (2016)[108]	Y	Y	Y	L	L	U	Y	U	U	U	Y	U	U	Y	Y	L
Nienhaus et al. (2011)[109]	Y	Y	U	L	L	U	Y	U	U	U	Y	U	U	U	U	U
Oren et al. (2016)[110]	Y	Y	Y	L	L	Y	Y	L	L	Y	Y	L	L	Y	Y	L
Pavic et al. (2015)[111]	Y	Y	Y	L	L	U	Y	U	U	U	Y	U	U	Y	Y	L
Reechaipichitkul et al. (2015).[112]	Y	Y	Y	L	L	U	Y	U	U	U	Y	U	U	Y	Y	L
Rose et al. (2015)[113]	Y	Y	Y	L	L	U	Y	U	U	U	Y	U	U	Y	Y	L
Salinas et al. (2015)[114]	Y	Y	Y	L	L	U	Y	U	U	U	Y	U	U	Y	Y	L
Sharma et al. (2017)[115]	Y	Y	Y	L	L	U	Y	U	U	U	Y	U	U	U	Y	U
Yoo et al. (2016)[116]	Y	Y	Y	L	L	U	Y	U	U	U	Y	U	U	U	Y	U
Borkowska et al. (2011)[117]	Y	Y	Y	L	L	U	Y	U	U	U	Y	U	U	U	Y	U

Balcells et al. (2008)[118]	Y	Y	Y	L	L	Y	Y	L	L	Y	Y	L	L	Y	Y	L
Bourgarit et al. (2015)[119]	Y	Y	Y	L	L	Y	Y	L	L	Y	Y	L	L	U	Y	U
Casas et al. (2011)[120]	Y	Y	Y	L	L	U	Y	U	U	U	Y	U	U	Y	Y	L
Casas et al. (2011)[121]	Y	Y	Y	L	L	U	Y	U	U	U	Y	U	U	Y	Y	L
Chkhartishvili et al. (2013)[122]	Y	Y	Y	L	L	U	Y	U	U	U	Y	U	U	U	Y	U
Gogus et al. (2010)[123]	Y	Y	Y	L	L	U	Y	U	U	U	Y	U	U	U	Y	U
Hanta et al. (2012)[124]	Y	Y	Y	L	L	U	Y	U	U	U	Y	U	U	U	Y	U
Hsia et al. (2012)[125]	Y	Y	Y	L	L	U	Y	U	U	U	Y	U	U	U	Y	U
James et al. (2014)[126]	Y	Y	Y	L	L	Y	Y	L	L	Y	Y	L	L	Y	Y	L
Jones et al. (2007)[127]	Y	Y	Y	L	L	U	Y	U	U	U	Y	U	U	U	Y	U
Karadag et al. (2010)[128]	Y	Y	Y	L	L	U	Y	U	U	U	Y	U	U	U	Y	U
Khawcharoenporn et al. (2015)[129]	Y	Y	Y	L	L	U	Y	U	U	U	Y	U	U	Y	Y	L
Kim et al. (2014)[130]	Y	Y	Y	L	L	U	Y	U	U	U	Y	U	U	U	Y	U
Kim et al. (2013)[131]	Y	Y	Y	L	L	U	Y	U	U	U	Y	U	U	U	Y	U
Kim et al. (2015)[132]	Y	Y	Y	L	L	U	Y	U	U	U	Y	U	U	Y	Y	L
Latorre et al. (2014)[133]	Y	Y	Y	L	L	U	Y	U	U	U	Y	U	U	U	Y	U
Manuel et al. (2007)[134]	Y	Y	Y	L	L	U	Y	U	U	U	Y	U	U	U	Y	U
Matulis et al. (2008)[135]	Y	Y	Y	L	L	Y	Y	L	L	Y	Y	L	L	U	Y	U
Mendez-Echevarria et al. (2011)[136]	Y	Y	Y	L	L	Y	Y	L	L	Y	Y	L	L	Y	Y	L
Papay et al. (2011)[137]	Y	Y	Y	L	L	Y	Y	L	L	Y	Y	L	L	Y	Y	L
Ramos et al. (2013)[138]	Y	Y	Y	L	L	Y	Y	L	L	Y	Y	L	L	Y	Y	L

Ramos et al. (2012)[139]	Y	Y	Y	L	L	Y	Y	L	L	Y	Y	L	L	Y	Y	L
Sauzullo et al. (2010)[140]	Y	Y	Y	L	L	Y	Y	L	L	Y	Y	L	L	Y	Y	L
Takahashi et al. (2007)[141]	Y	Y	Y	L	L	U	Y	U	U	U	Y	U	U	U	Y	U
Vassilopoulos et al. (2011)[142]	Y	Y	Y	L	L	Y	Y	L	L	Y	Y	L	L	U	Y	U
Hoffmann et al. (2010)[143]	Y	Y	Y	L	L	U	Y	U	U	U	Y	U	U	Y	Y	L
Mariette et al. (2012)[144]	Y	Y	Y	L	L	Y	Y	L	L	Y	Y	L	L	Y	Y	L
Ponce de Leon et al. (2008)[145]	Y	Y	Y	L	L	U	Y	U	U	U	Y	U	U	U	Y	U
Scrivo et al. (2012)[146]	Y	Y	Y	L	L	Y	Y	L	L	Y	Y	L	L	Y	Y	L
Cho et al. (2016)[147]	Y	Y	Y	L	L	U	Y	U	U	U	Y	U	U	U	Y	U
Kurti et al. (2015)[148]	Y	Y	Y	L	L	Y	Y	L	L	Y	Y	L	L	U	Y	U
Kussen et al. (2016)[149]	Y	Y	Y	L	L	U	Y	U	U	U	Y	U	U	U	Y	U
Palomar et al. (2011)[150]	Y	Y	Y	L	L	U	Y	U	U	U	Y	U	U	Y	Y	L
Anibarro et al. (2011)[151]	Y	Y	Y	L	L	Y	Y	L	L	Y	Y	L	L	Y	Y	L
Diel et al. (2008)[152]	Y	Y	Y	L	L	Y	Y	L	L	Y	Y	L	L	Y	Y	L
Ferreira et al. (2015)[153]	Y	Y	Y	L	L	Y	Y	L	L	Y	Y	L	L	Y	Y	L
Nienhaus et al. (2008)[154]	Y	Y	Y	L	L	Y	Y	L	L	Y	Y	L	L	Y	Y	L
Minguez et al. (2012)[155]	Y	Y	Y	L	L	Y	Y	L	L	Y	Y	L	L	U	Y	U
Moon et al. (2013)[156]	Y	Y	Y	L	L	Y	Y	L	L	Y	Y	L	L	Y	Y	L
Talati et al. (2009)[157]	Y	Y	Y	L	L	Y	Y	L	L	Y	Y	L	L	Y	Y	L

References

1. Diel R, Nienhaus A, Lange C, Meywald-Walter K, Forssbohm M, Schaberg T. Tuberculosis contact investigation with a new, specific blood test in a low-incidence population containing a high proportion of BCG-vaccinated persons. Respir Res. 2006; 7: 77.
2. Nakaoka H, Lawson L, Squire BS, Coulter B, Ravn P, Brock I, et al. Risk for tuberculosis among children. Emerg Infect Dis. 2006; 12: 1383–1388.
3. Porsa E, Cheng L, Seale MM, Delclos GL, Ma X, Reich R, et al. Comparison of a new ESAT-6/CFP-10 peptide-based gamma interferon assay and a tuberculin skin test for tuberculosis screening in a moderate-risk population. Clin Vaccine Immunol. 2006; 13: 53-8.
4. Tsiouris SJ, Austin J, Toro P, Coetzee D, Weyer K, Stein Z, et al. Results of a tuberculosis-specific IFN-gamma assay in children at high risk for tuberculosis infection. Int J Tuberc Lung Dis. 2006; 10: 939-941.
5. Adetifa IM, Lugos MD, Hammond A, Jeffries D, Donkor S, Adegbola RA, et al. Comparison of two interferon gamma release assays in the diagnosis of Mycobacterium tuberculosis infection and disease in The Gambia. BMC Infect Dis. 2007; 7: 122.
6. Arend SM, Thijsen SF, Leyten EM, Bouwman JJ, Franken WP, Koster BF, et al. Comparison of two interferon-gamma assays and tuberculin skin test for tracing tuberculosis contacts. Am J Respir Crit Care Med. 2007; 175: 618-627.
7. Dogra S, Narang P, Mendiratta DK, Chaturvedi P, Reingold AL, Colford JM, et al. Comparison of a whole blood interferon-gamma assay with tuberculin skin testing

for the detection of tuberculosis infection in hospitalized children in rural India. J Infect. 2007; 54: 267-276.

8. Franken WP, Timmermans JF, Prins C, Slootman EJ, Dreverman J, Bruins H, et al. Comparison of Mantoux and Quanti-FERON TB gold tests for diagnosis of latent tuberculosis infection in army personnel. Clin Vaccine Immunol. 2007; 14: 477-480.

9. Rangaka MX, Wilkinson KA, Seldon R, Van Cutsem G, Meintjes GA, Morroni G, et al. Effect of HIV-1 infection on T-Cell-based and skin test detection of tuberculosis infection. Am J Respir Crit Care Med. 2007; 175: 514-20.

10. Silverman MS, Reynolds D, Kavsak PA, Garay J, Daly A, Davis I. Use of an interferon-gamma based assay to assess bladder cancer patients treated with intravesical BCG and exposed to tuberculosis. Clin Biochem. 2007; 40: 913-915.

11. Chun JK, Kim CK, Kim HS, Jung GY, Lee TJ, Kim KH, et al. The role of a whole blood interferon-gamma assay for the detection of latent tuberculosis infection in Bacille Calmette-Guerin vaccinated children. Diagn Microbiol Infect Dis. 2008; 62: 389-394.

12. Bienek DR and Chang CK. Evaluation of an interferon-gamma release assay, T-SPOT.TB, in a population with a low prevalence of tuberculosis. Int J Tuberc Lung Dis. 2009. 13: 1416-21.

13. Janssens JP, Lombard PR, Perneger T, Metzger M, Vivien R, Rochat T. Contribution of a IFN-gamma assay in contact tracing for tuberculosis in a low-incidence, high immigration area. Swiss Med Wkly. 2008; 138: 585-93.

14. Leung CC, Yam WC, Yew WW, Ho PL, Tam CM, Law WS, et al. Comparison of T-Spot.TB and tuberculin skin test among silicotic patients. Eur Respir J. 2008; 31: 266-72.

15. Mirtskhulava V, Kempker R, Shields KL, Leonard MK, Tsertsvadze T, del Rio C, et al. Prevalence and risk factors for latent tuberculosis infection among health care workers in Georgia. *Int J Tuberc Lung Dis.* 2008; 12: 513-519.

16. Petrucci R, Abu Amer N, Gurgel RQ, Sherchand JB, Doria L, Lama C, et al. Interferon gamma, interferon-gamma-induced-protein 10, and tuberculin responses of children at high risk of tuberculosis infection. Pediatr Infect Dis J. 2008; 27: 1073-1077.

17. Soysal A, Torun T, Efe S, Gencer H, Tahaoglu K, Bakir M. Evaluation of cut-off values of interferon-gamma-based assays in the diagnosis of M. tuberculosis infection. Int J Tuberc Lung Dis. 2008; 12: 50-6.

18. Baker CA, Thomas W, Stauffer WM, Peterson PK, Tsukayama DT. Serial testing of refugees for latent tuberculosis using the QuantiFERON-gold in-tube: effects of an antecedent tuberculin skin test. Am J Trop Med Hyg. 2009; 80: 628-633.

19. Bianchi L, Galli L, Moriondo M, Veneruso G, Becciolini L, Azzari C, et al. Interferon-gamma release assay improves the diagnosis of tuberculosis in children. Pediatr Infect Dis J. 2009; 28: 510-514.

20. Fox BD, Kramer MR, Mor Z, Preiss R, Rusanov V, Fuks L, et al. The QuantiFERON-TB-GOLD assay for tuberculosis screening in healthcare workers: a cost-comparison analysis. Lung. 2009; 187: 413-419.

21. Girardi E, Angeletti C, Puro V, Sorrentino R, Magnavita N, Vincenti D, et al. Estimating diagnostic accuracy of tests for latent tuberculosis infection without a gold standard among healthcare workers. Euro Surveill. 2009; 14.

22. Hansted E, Andriuskeviciene A, Sakalauskas R, Kevalas R, Sitkauskiene B. T-cell-based diagnosis of tuberculosis infection in children in Lithuania: a country of high

incidence despite a high coverage with bacille Calmette-Guerin vaccination. BMC Pulm Med. 2009; 9: 41.

23. Herrmann JL, Simonney N, Bergeron A, Ducreux-Adolphe N, Porcher R, Rouveau M, et al. IFN-gamma and antibody responses among French nurses during a tuberculosis contact tracing investigation. Pathol Biol (Paris). 2009; 57: e49-53.

24. Kik SV, Franken WP, Arend SM, Mensen M, Cobelens FG, Kamphorst M, et al. Interferon-gamma release assays in immigrant contacts and effect of remote exposure to Mycobacterium tuberculosis. Int J Tuberc Lung Dis. 2009; 13: 820-828.

25. Kim EY, Lim JE, Jung JY, Son JY, Lee KJ, Yoon YW, et al. Performance of the tuberculin skin test and interferon-gamma release assay for detection of tuberculosis infection in immunocompromised patients in a BCG-vaccinated population. BMC Infect Dis. 2009; 9: 207.

26. Lien LT, Hang NT, Kobayashi N, Yanai H, Toyota E, Sakurada S, et al. Prevalence and risk factors for tuberculosis infection among hospital workers in Hanoi, Viet Nam. PloS One. 2009; 4: e6798.

27. Lighter J, Rigaud M, Eduardo R, Peng CH, Pollack H. Latent tuberculosis diagnosis in children by using the QuantiFERON-TB Gold In-Tube test. Pediatrics. 2009; 123: 30-37.

28. Machado A Jr, Emodi K, Takenami I, Finkmoore BC, Barbosa T, Carvalho J, et al. Analysis of discordance between the tuberculin skin test and the interferon-gamma release assay. Int J Tuberc Lung Dis. 2009; 13: 446-453.

29. Ringshausen FC, Schlosser S, Nienhaus A, Schablon A, Schultze-Werninghaus G, Rohde G. In-hospital contact investigation among health care workers after exposure to smear-negative tuberculosis. J Occup Med Toxicol. 2009; 4: 11.

30. Saracino A, Scotto G, Fornabaio C, Martinelli D, Faleo G, Cibelli D, et al. QuantiFERON-TB Gold In-Tube test (QFT-GIT) for the screening of latent tuberculosis in recent immigrants to Italy. The New Microbiologica. 2009; 32: 369-376.
31. Torres Costa J, Sa R, Cardoso MJ, Silva R, Ferreira J, Ribeiro C, et al. Tuberculosis screening in Portuguese healthcare workers using the tuberculin skin test and the interferon-gamma release assay. Eur Respir J. 2009; 34: 1423-1428.
32. Tripodi D, Brunet-Courtois B, Nael V, Audrain M, Chailleux E, Germaud P, et al. Evaluation of the tuberculin skin test and the interferon-γ release assay for TB screening in French healthcare workers. J Occup Med Toxicol. 2009; 4: 30.
33. Vinton P, Mihrshahi S, Johnson P, Jenkin GA, Jolley D, Biggs BA. Comparison of QuantiFERON-TB Gold In-Tube Test and tuberculin skin test for identification of latent Mycobacterium tuberculosis infection in healthcare staff and association between positive test results and known risk factors for infection. Infect Control Hosp Epidemiol. 2009; 30: 215-221.
34. Zhao X, Mazlagic D, Flynn EA, Hernandez H, Abbott CL. Is the QuantiFERON-TB blood assay a good replacement for the tuberculin skin test in tuberculosis screening? a pilot study at Berkshire Medical Center. Am J Clin Pathol. 2009; 132: 678-686.
35. Adetifa IM, Ota MO, Jeffries DJ, Hammond A, Lugos MD, Donkor S, et al. Commercial interferon gamma release assays compared to the tuberculin skin test for diagnosis of latent Mycobacterium tuberculosis infection in childhood contacts in the Gambia. Pediatr Infect Dis J. 2010; 29: 439-443.
36. Costa JT, Silva R, Sa R, Cardoso MJ, Ribeiro C, Nienhaus A. Comparison of interferon-gamma release assay and tuberculin test for screening in healthcare workers. Rev Port Pneumol. 2010; 16: 211-221.

37. Grare, M., et al., QuantiFERON-TB Gold In-Tube as help for the diagnosis of tuberculosis in a French pediatric hospital. Diagn Microbiol Infect Dis, 2010. 66(4): p. 366-72.

38. Huang, Y.W., et al., Latent tuberculosis infection among close contacts of multidrug-resistant tuberculosis patients in central Taiwan. Int J Tuberc Lung Dis, 2010. 14(11): p. 1430-5.

39. Jong Lee, K., et al., Screening for latent tuberculosis infection in South Korean healthcare workers using a tuberculin skin test and whole blood interferon-gamma assay. Scand J Infect Dis, 2010. 42(9): p. 672-8.

40. Katsenos, S., et al., Interferon-gamma release assay clarifies the effect of bacille Calmette-Guerin vaccination in Greek army recruits. Int J Tuberc Lung Dis, 2010. 14(5): p. 545-50.

41. Lee, S.H., et al., Serial interferon-gamma release assays after rifampicin prophylaxis in a tuberculosis outbreak. Respir Med, 2010. 104(3): p. 448-53.

42. Leung, C.C., et al., T-Spot.TB outperforms tuberculin skin test in predicting tuberculosis disease. Am J Respir Crit Care Med, 2010. 182(6): p. 834-40.

43. Torres Costa, J., et al., Results of five-year systematic screening for latent tuberculosis infection in healthcare workers in Portugal. J Occup Med Toxicol, 2010. 5: p. 22.

44. Thomas, T.A., et al., Malnutrition and helminth infection affect performance of an interferon gamma-release assay. Pediatrics, 2010. 126(6): p. e1522-9.

45. Tsolia, M.N., et al., Whole blood interferon-gamma release assay is a useful tool for the diagnosis of tuberculosis infection particularly among Bacille Calmette Guerin-vaccinated children. Pediatr Infect Dis J, 2010. 29(12): p. 1137-40.

46. Caglayan V, Ak O, Dabak G, Damadoglu E, Ketenci B, Ozdemir M, et al. Comparison of tuberculin skin testing and QuantiFERON-TB Gold-In Tube test in health care workers. Tuberk Toraks. 2011; 59: 43-47.

47. Diel R, Loddenkemper R, Niemann S, Meywald-Walter K, Nienhaus A. Negative and positive predictive value of a whole-blood interferon-gamma release assay for developing active tuberculosis: an update. Am J Respir Crit Care Med. 2011; 183: 88-95.

48. Kasambira TS, Shah M, Adrian PV, Holshouser M, Madhi SA, Chaisson RE, et al. QuantiFERON-TB Gold In-Tube for the detection of Mycobacterium tuberculosis infection in children with household tuberculosis contact. Int J Tuberc Lung Dis. 2011; 15: 628-634.

49. Kus J, Demkow U, Lewandowska K, Korzeniewska-Kosela M, Rabczenko D, Siemion-Szczesniak I, et al. Prevalence of latent infection with Mycobacterium tuberculosis in Mazovia Region using interferon gamma release assay after stimulation with specific antigens ESAT-6 and CFP-10. Pneumonologia i Alergologia Polska. 2011; 79: 407-418.

50. Legesse M, Ameni G, Mamo G, Medhin G, Bjune G, Abebe F. Community-based cross-sectional survey of latent tuberculosis infection in Afar pastoralists, Ethiopia, using QuantiFERON-TB Gold In-Tube and tuberculin skin test. BMC Infect Dis. 2011; 11: 89.

51. Moon HW, Kim H, Hur M, Yun YM, Lee A. Latent tuberculosis infection screening for laboratory personnel using interferon-gamma release assay and tuberculin skin test in Korea: an intermediate incidence setting. J Clin Lab Anal. 2011; 25: 382-388.

52. Moyo S, Isaacs F, Gelderbloem S, Verver S, Hawkridge AJ, Hatherill M, et al. Tuberculin skin test and QuantiFERON(R) assay in young children investigated for tuberculosis in South Africa. Int J Tuberc Lung Dis. 2011; 15: 1176-1181.

53. Pavic I, Topic RZ, Raos M, Aberle N, Dodig S. Interferon-gamma release assay for the diagnosis of latent tuberculosis in children younger than 5 years of age. Pediatr Infect Dis J. 2011; 30: 866-870.

54. Rafiza S RK, Tahir A. Prevalence and risk factors of latent tuberculosis infection among health care workers in Malaysia. BMC Infect Dis. 2011; 11: 19.

55. Shanaube K, Hargreaves J, Fielding K, Schapp A, Lawrence KA, Hensen B, et al. Risk factors associated with positive QuantiFERON-TB Gold In-Tube and tuberculin skin tests results in Zambia and South Africa. PloS One. 2011; 6: e18206.

56. Talebi-Taher M, Javad-Moosavi SA, Entezari AH, Shekarabi M, Parhizkar B. Comparing the performance of QuantiFERON-TB Gold and Mantoux test in detecting latent tuberculosis infection among Iranian health care workers. Int J Occup Med Environ Health. 2011; 24: 359-366.

57. Torres Costa J, Silva R, Ringshausen FC, Nienhaus A. Screening for tuberculosis and prediction of disease in Portuguese healthcare workers. J Occup Med Toxicol. 2011; 6: 19.

58. Torres Costa J, Silva R, Sa R, Cardoso M, Nienhaus A. Serial testing with the interferon-gamma release assay in Portuguese healthcare workers. Int Arch Occup Environ Health. 2011; 84: 461-469.

59. Weinfurter P, Blumberg HM, Goldbaum G, Royce R, Pang J, Tapia J, t al. Predictors of discordant tuberculin skin test and QuantiFERON(R)-TB Gold In-Tube results in various high-risk groups. Int J Tuberc Lung Dis. 2011; 15: 1056-1061.

60. Yassin MA, Petrucci R, Garie KT, Harper G, Arbide I, Aschalew M, et al. Can interferon-gamma or interferon-gamma-induced-protein-10 differentiate tuberculosis infection and disease in children of high endemic areas? PloS One. 2011; 6: e23733.

61. Zhao J, Wang Y, Wang H, Jiang C, Liu Z, Meng X, et al. Low agreement between the T-SPOT(R).TB assay and the tuberculin skin test among college students in China. Int J Tuberc Lung Dis. 2011; 15: 134-6.

62. Bergot E, Haustraete E, Malbruny B, Magnier R, Salaun MA, Zalcman G. Observational study of QuantiFERON(R)-TB gold in-tube assay in tuberculosis contacts in a low incidence area. PloS One. 2012; 7: e43520.

63. Di Renzi S, Tomao P, Martini A, Capanna S, Rubino L, D'Amico W, et al. Screening for tuberculosis among homeless shelter staff. Am J Infect Control. 2012; 40: 459-461.

64. He GX, Wang LX, Chai SJ, Klena JD, Cheng SM, Ren YL, et al. Risk factors associated with tuberculosis infection among health care workers in Inner Mongolia, China. Int J Tuberc Lung Dis. 2012; 16: 1485-1491.

65. Jeong YJ, Yoon S, Koo HK, Lim HJ, Lee JS, Lee SM, et al. Positive tuberculin skin test or interferon-gamma release assay in patients with radiographic lesion suggesting old healed tuberculosis. J Korean Med Sci. 2012; 27: 761-766.

66. Jo KW, Jeon K, Kang YA, Koh WJ, Kim KC, Kim YH, et al. Poor correlation between tuberculin skin tests and interferon-gamma assays in close contacts of patients with multidrug-resistant tuberculosis. Respirology. 2012; 17: 1125-1130.

67. Jung da H, Jo KW, Shim TS. Prevalence of latent tuberculosis infection among medical students in South Korea. Tuberc Resp Dis. 2012; 73: 219-223.

68. Larcher C, Frizzera E, Pretto P, Lang M, Sonnleitner N, Huemer HP. Immunosurveillance for Mycobacterium tuberculosis of health care personnel in a third level care hospital. Med Lav. 2012; 103: 26-36.

69. Nkurunungi G, Lutangira JE, Lule SA, Akurut H, Kizindo R, Fitchett JR, et al. Determining Mycobacterium tuberculosis infection among BCG-immunised Ugandan children by T-SPOT.TB and tuberculin skin testing. PLoS One. 2012; 7: e47340.

70. Onur H, Hatipoglu S, Arica V, Hatipoglu N, Arica SG. Comparison of quantiferon test with tuberculin skin test for the detection of tuberculosis infection in children. Inflammation. 2012; 35: 1518-1524.

71. Pattnaik S, John KR, Shalini E, Michael JS. Agreement between skin testing and QuantiFERON-TB Gold In-Tube assay (QFT-TB) in detecting latent tuberculosis infection among household contacts in India. Indian J Tuberc. 2012; 59: 214-218.

72. Zwerling A, Cojocariu M, McIntosh F, Pietrangelo F, Behr MA, Schwartzman K, et al. TB screening in Canadian health care workers using interferon-gamma release assays. PloS One. 2012; 7: e43014.

73. Jo KW, Hong Y, Park JS, Bae IG, Eom JS, Lee SR, et al. Prevalence of latent tuberculosis infection among health care workers in South Korea: a multicenter study. Tuberc Resp Dis. 2013; 75: 18-24.

74. Serrano-Escobedo CJ, Enciso-Moreno JA, Monarrez-Espino J. Performance of tuberculin skin test compared to QFT-IT to detect latent TB among high-risk contacts in Mexico. Arch Med Res. 2013; 44: 242-248.

75. Whitaker JA, Mirtskhulava V, Kipiani M, Harris DA, Tabagari N, Kempker RR, et al. Prevalence and incidence of latent tuberculosis infection in georgian healthcare workers. PloS One. 2013; 8: e58202.

76. Zwerling A, Benedetti A, Cojocariu M, McIntosh F, Pietrangelo F, Behr MA, et al. Repeat IGRA testing in Canadian health workers: conversions or unexplained variability? PloS One. 2013; 8: e54748.

77. Aichelburg MC, Mandorfer M, Tittes J, Breitenecker F, Reiberger T, Rieger A, et al. The association of smoking with IGRA and TST results in HIV-1-infected subjects. Int J Tuberc Lung Dis. 2014; 18: 709-16.

78. Alvarez GG, Van Dyk DD, Davies N, Aaron SD, Cameron DW, Desjardings M, et al. The feasibility of the interferon gamma release assay and predictors of discordance with the tuberculin skin test for the diagnosis of latent tuberculosis infection in a remote Aboriginal community. PloS One. 2014; 9: e111986.

79. Charisis A, Tatsioni A, Gartzonika C, Gogali A, Archimandriti D, Katsanos C, et al. Value of adding an IGRA to the TST to screen for latent tuberculous infection in Greek health care workers. Int J Tuberc Lung Dis. 2014; 18: 1040-1046.

80. de Souza FM, do Prado TN, Pinheiro Jdos S, Peres RL, Lacerda TC, Loureiro RB, et al. Comparison of interferon-gamma release assay to two cut-off points of tuberculin skin test to detect latent Mycobacterium tuberculosis infection in primary health care workers. PloS One. 2014; 9: e102773.

81. Erkens CG, Dinmohamed AG, Kamphorst M, Toumanian S, van Nispen-Dobrescu R, Alink M, et al. Added value of interferon-gamma release assays in screening for tuberculous infection in the Netherlands. Int J Tuberc Lung Dis. 2014; 18: 413-420.

82. Garazzino S, Galli L, Chiappini E, Pinon M, Bergamini BM, Cazzato S, et al. Performance of interferon-gamma release assay for the diagnosis of active or latent tuberculosis in children in the first 2 years of age: a multicenter study of the Italian Society of Pediatric Infectious Diseases. Pediatr Infect Dis J. 2014; 33: e226-231.

83. Guanche Garcell H, Crespo Ramirez E, Kindelan Contreras A, Gutierrez Garcia F. Latent tuberculosis infection in healthcare workers at a community hospital in Qatar. J Infect Public Health. 2014; 7: 356-359.

84. Goodwin DJ, Mazurek GH, Campbell BH, Bohanon J, West KB, Bell JJ, et al. Automation of an interferon-gamma release assay and comparison to the tuberculin skin test for screening basic military trainees for Mycobacterium tuberculosis infection. Mil Med. 2014; 179: 333-341.

85. Mathad JS, Bhosale R, Sangar V, Mave V, Gupte N, Kanade S, et al. Pregnancy differentially impacts performance of latent tuberculosis diagnostics in a high-burden setting. PloS One. 2014; 9: e92308.

86. Ribeiro-Rodrigues R, Kim S, Coelho da Silva FD, Uzelac A, Collins L, Palaci M, et al. Discordance of tuberculin skin test and interferon gamma release assay in recently exposed household contacts of pulmonary TB cases in Brazil. PloS One. 2014; 9: e96564.

87. Sauzullo I, Mastroianni CM, Mengoni F, Ermocida A, Mascia C, Salotti A, et al. Long-term IFN-gamma and IL-2 response for detection of latent tuberculosis infection in healthcare workers with discordant immunologic results. J Immunol Methods. 2014; 414: 51-57.

88. Song SE, Yang J, Lee KS, Kim H, Kim YM, Kim S, et al. Comparison of the tuberculin skin test and interferon gamma release assay for the screening of tuberculosis in adolescents in close contact with tuberculosis TB patients. PloS One. 2014; 9: e100267.

89. Adams S, Ehrlich R, Baatjies R, van Zyl-Smit RN, Said-Hartley Q, Dawson R, et al. Incidence of occupational latent tuberculosis infection in South African healthcare workers. Eur Respir J. 2015; 45: 1364-1373.

90. El-Sokkary RH, Abu-Taleb AM, El-Seifi OS, Zidan HE, Mortada EM, El-Hossary D, et al. Assessing the Prevalence of Latent Tuberculosis among Health Care Providers in Zagazig City, Egypt Using Tuberculin Skin Test and QuantiFERON-TB Gold In-Tube Test. Cent Eur J Public Health. 2015; 23: 324-330.

91. Gao L, Lu W, Bai L, Wang X, Xu J, Catanzaro A, et al. Latent tuberculosis infection in rural China: baseline results of a population-based, multicentre, prospective cohort study. Lancet Infect Dis. 2015; 15: 310-319.

92. Goebel KM, Tay EL, Denholm JT. Supplemental use of an interferon-gamma release assay in a state-wide tuberculosis contact tracing program in Victoria: a six-year review. Commun Dis Intell Q Rep. 2015; 39: E191-196.

93. He G, Li Y, Zhao F, Wang L, Cheng S, Guo H, et al. The prevalence and incidence of latent tuberculosis infection and its associated factors among village doctors in China. PloS One. 2015; 10: e0124097.

94. Howley MM, Painter JA, Katz DJ, Graviss EA, Reves R, Beavers SF, et al. Evaluation of QuantiFERON-TB gold in-tube and tuberculin skin tests among immigrant children being screened for latent tuberculosis infection. Pediatr Infect Dis J. 2015; 34: 35-39.

95. Jones-Lopez EC, White LF, Kirenga B, Mumbowa F, Ssebidandi M, Moine S, et al. Cough aerosol cultures of Mycobacterium tuberculosis: insights on TST / IGRA discordance and transmission dynamics. PloS One. 2015; 10: e0138358.

96. Leung CC, Yam WC, Ho PL, Yew WW, Chan CK, Law WS, et al. T-Spot.TB outperforms tuberculin skin test in predicting development of active tuberculosis among household contacts. Respirology. 2015; 20: 496-503.

97. Spicer KB, Turner J, Wang SH, Koranyi K, Powell DA. Tuberculin skin testing and T-SPOT.TB in internationally adopted children. Pediatr Infect Dis J. 2015; 34: p. 599-603.

98. Lucet JC, Abiteboul D, Estellat C, Roy C, Chollet-Martin S, Tubach F, et al. Interferon-gamma release assay vs. tuberculin skin test for tuberculosis screening in exposed healthcare workers: a longitudinal multicenter comparative study. Infect Control Hosp Epidemiol. 2015; 36: 569-574.

99. Ferrarini MA, Spina FG, Weckx LY, Lederman HM, De Moraes-Pinto MI. Rate of tuberculosis infection in children and adolescents with household contact with adults with active pulmonary tuberculosis as assessed by tuberculin skin test and interferon-gamma release assays. Epidemiol Infect. 2016; 144: 712-723.

100. Al Hajoj S, Varghese B, Datijan A, Shoukri M, Alzahrani A, Alkhenizan A, et al. Interferon gamma release assay versus tuberculin skin testing among healthcare workers of highly diverse origin in a moderate tuberculosis burden country. PLoS One. 2016; 11: e0154803.

101. Biraro IA, Kimuda S, Egesa M, Cose S, Webb EL, Joloba M, et al. The use of interferon gamma inducible protein 10 as a potential biomarker in the diagnosis of latent tuberculosis infection in Uganda. PLoS One. 2016; 11: e0146098.

102. Bozkanat E, Kaya H, Sezer O, Caliskan T, Kilic E, Ciftci F, et al. Comparison of tuberculin skin test and quantiferon-TB gold in tube test for diagnosis of latent

tuberculosis infection in health care workers: A cross sectional study. J Pak Med Assoc. 2016; 66: 270-4.

103. Grare M, Derelle J, Dailloux M, Laurain C. Difficulties of TB diagnosis in children: QuantiFERON TB Gold In-Tube as useful tool. Arch Pediatr. 2010; 17: 77-85.

104. Lowenthal P, Barry PM, Flood J. High discordance between pre-US and post-US entry tuberculosis test results among immigrant children: is it time to adopt interferon gamma release assay for pre-entry tuberculosis screening? Pediatr Infect Dis J. 2016; 35: 231-6.

105. Marco Mourino A, Orcau Palau A, Jane Galliga R, Escribano Ibanez M, Cayla Buqueras JA, Sole Zapata N, et al. Concordance of tuberculin tests and Interferon gamma release assays in the prison population. Rev Esp Sanid Penit. 2011; 13: 15-20.

106. Marquez C, Chamie G, Achan J, Luetkemeyer A, Kyohere M, Okiring J, et al. Tuberculosis infection in early childhood and the association with HIV-exposure in HIV-uninfected children in rural uganda. Pediatr Infect Dis J. 2016; 35: 524-9.

107. Miramontes R, Hill AN, Woodruff RSY, Lambert LA, Navin TR, Castro KG, et al. Tuberculosis infection in the United States: prevalence estimates from the National Health and Nutrition Examination Survey, 2011-2012. PLoS One. 2015; 10: e0140881.

108. Mostafavi E, Nasehi M, Shahraki AH, Esmaeili S, Ghaderi E, Sharafi S, et al. Comparison of the tuberculin skin test and the QuantiFERON-TB Gold test in detecting latent tuberculosis in health care workers in Iran. Epidemiol Health. 2016; 38: e2016032.

109. Nienhaus A, Schablon A, Tripodi D, Torres Costa J. The prevalence of latent tuberculosis infections among health-care workers--a three-country comparison. Pneumologie. 2011; 65: 726-9.

110. Oren E, Fiero MH, Barrett E, Anderson B, Nunez M, Gonzalez-Salazar F. Detection of latent tuberculosis infection among migrant farmworkers along the US-Mexico border. BMC Infect Dis. 2016; 16: 630.

111. Pavic I, Katalinic-Jankovic V, Cepin-Bogovic J, Resic A, Dodig S. Discordance between tuberculin skin test and interferon-gamma release assay in children younger than 5 years who have been vaccinated with Bacillus Calmette-Guerin. Lab Med. 2015; 46: 200-6.

112. Reechaipichitkul W, Pimrin W, Bourpoern J, Prompinij S, Faksri K. Evaluation of the QuantiFERON-TB Gold In-Tube assay and tuberculin skin test for the diagnosis of Mycobacterium tuberculosis infection in northeastern Thailand. Asian Pac J Allergy Immunol. 2015; 33: 236-44.

113. Rose W, Read SE, Bitnun A, Rea E, Stephens D, Pongsamart W. Relating tuberculosis (TB) contact characteristics to QuantiFERON-TB-Gold and tuberculin skin test results in the Toronto pediatric TB clinic. J Pediatric Infect Dis Soc. 2015; 4: 96-103.

114. Salinas C, Ballaz A, Diez R, Aguirre U, Anton A, Altube L. Tuberculosis screening program for undocumented immigrant teenagers using the QuantiFERON((R))-TB Gold In-Tube test. Med Clin (Barc). 2015; 145: 7-13.

115. Sharma SK, Vashishtha R, Chauhan LS, Sreenivas V, Seth D. Comparison of TST and IGRA in diagnosis of latent tuberculosis infection in a high TB-burden setting. PLoS One. 2017; 12: e0169539.

116. Yoo JW, Jo KW, Park GY, Shim TS. Comparison of latent tuberculosis infection rate between contacts with active tuberculosis and non-contacts. Respir Med. 2016; 111: 77-83.

117. Borkowska D, Zwolska Z, Michałowska-Mitczuk D, Korzeniewska-Koseła M, Zabost A, Napiórkowska A, et al. Interferon-gamma assays T-SPOT.TB for the diagnosis of latent tuberculosis infection. Pneumonol Alergol Pol. 2011; 79: 264-71.

118. Balcells ME, Perez CM, Chanqueo L, Lasso M, Villanueva M, Espinoza M, et al. A comparative study of two different methods for the detection of latent tuberculosis in HIV-positive individuals in Chile. Int J Infect Dis. 2008; 12: 645-52.

119. Bourgarit A, Baron G, Breton G, Tattevin P, Katlama C, Allavena C, et al. Latent tuberculosis infection screening and 2-year outcome in antiretroviral-naive HIV-infected patients in a low-prevalence country. Ann Am Thorac Soc. 2015; 12: 1138-45.

120. Casas S, Andreu A, Juanola X, Bordas X, Alcaide F, Moure R, et al. Diagnosis of tuberculosis infection by tuberculin skin test and a whole-blood interferon-gamma release assay in patients considered for anti-tumor necrosis factor-alpha therapy. Diagn Microbiol Infect Dis. 2011; 71: 57-65.

121. Casas S, Muñoz L, Moure R, Castellote J, Guerra MR, Gonzalez L, et al. Comparison of the 2-step tuberculin skin test and the quantiFERON-TB Gold In-Tube Test for the screening of tuberculosis infection before liver transplantation. Liver Transpl. 2011; 17: 205-11.

122. Chkhartishvili N, Kempker RR, Dvali N, Abashidze L, Sharavdze L, Gabunia P, et al. Poor agreement between interferon-gamma release assays and the tuberculin skin test among HIV-infected individuals in the country of Georgia. BMC Infect Dis. 2013; 13: 513.

123. Gogus F, Gunendi Z, Karakus R, Erdogan Z, Hizel K, Atalay F. Comparison of tuberculin skin test and QuantiFERON-TB gold in tube test in patients with chronic

inflammatory diseases living in a tuberculosis endemic population. Clin Exp Med. 2010; 10: 173-7.

124. Hanta I, Ozbek S, Kuleci S, Seydaoglu G, Ozyilmaz E. Detection of latent tuberculosis infection in rheumatologic diseases before anti-TNFalpha therapy: tuberculin skin test versus IFN-gamma assay. Rheumatol Int. 2012; 32: 3599-603.

125. Hsia EC, Schluger N, Cush JJ, Chaisson RE, Matteson EL, Xu S, et al. Interferon-gamma release assay versus tuberculin skin test prior to treatment with golimumab, a human anti-tumor necrosis factor antibody, in patients with rheumatoid arthritis, psoriatic arthritis, or ankylosing spondylitis. Arthritis Rheum. 2012; 64: 2068-77.

126. James PM, Ganaie FA, Kadahalli RL. The performance of quantiferon-TB gold in-tube (QFT-IT) test compared to tuberculin skin test (TST) in detecting latent tuberculosis infection (LTBI) in the presence of HIV coinfection in a high TB-burden area with BCG-vaccinated population. J Int Assoc Provid AIDS Care. 2014; 13: 47-55.

127. Jones S, de Gijsel D, Wallach FR, Gurtman AC, Shi Q, Sacks H. Utility of QuantiFERON-TB Gold in-tube testing for latent TB infection in HIV-infected individuals. Int J Tuberc Lung Dis. 2007; 11: 1190-5.

128. Karadag O, Aksu K, Sahin A, Zihni FY, Sener B, Inanc N, et al. Assessment of latent tuberculosis infection in Takayasu arteritis with tuberculin skin test and Quantiferon-TB Gold test. Rheumatol Int. 2010; 30: 1483-7.

129. Khawcharoenporn T, Apisarnthanarak A, Phetsuksiri B, Rudeeaneksin J, Srisungngam S, Mundy LM. Tuberculin skin test and QuantiFERON-TB Gold In-tube Test for latent tuberculosis in Thai HIV-infected adults. Respirology. 2015; 20: 340-7.

130. Kim HC, Jo KW, Jung YJ, Yoo B, Lee CK, Kim YG, et al. Diagnosis of latent tuberculosis infection before initiation of anti-tumor necrosis factor therapy using both tuberculin

skin test and QuantiFERON-TB Gold In Tube assay. Scand J Infect Dis. 2014; 46: 763-9.

131. Kim JH, Cho SK, Han M, Choi CB, Kim TH, Jun JB, et al. Factors influencing discrepancies between the QuantiFERON-TB gold in tube test and the tuberculin skin test in Korean patients with rheumatic diseases. Semin Arthritis Rheum. 2013; 42: 424-32.

132. Kim JH, Won S, Choi CB, Sung YK, Song GG, Bae SC. Evaluation of the usefulness of interferon-gamma release assays and the tuberculin skin test for the detection of latent Mycobacterium tuberculosis infections in Korean rheumatic patients who are candidates for biologic agents. Int J Rheum Dis. 2015; 18: 315-22.

133. Latorre I, Carrascosa JM, Vilavella M, Díaz J, Prat C, Domínguez J, et al. Diagnosis of tuberculosis infection by interferon-gamma release assays in patients with psoriasis. J Infect. 2014; 69: 600-6.

134. Manuel O, Humar A, Preiksaitis J, Doucette K, Shokoples S, Peleg AY, et al. Comparison of quantiferon-TB gold with tuberculin skin test for detecting latent tuberculosis infection prior to liver transplantation. Am J Transplant. 2007; 7: 2797-801.

135. Matulis G, Jüni P, Villiger PM, Gadola SD. Detection of latent tuberculosis in immunosuppressed patients with autoimmune diseases: performance of a Mycobacterium tuberculosis antigen-specific interferon gamma assay. Ann Rheum Dis. 2008; 67: 84-90.

136. Mendez-Echevarria A, Gonzalez-Munoz M, Mellado MJ, Baquero-Artigao F, Vecino R, Perez E. Optimizing interpretation of the tuberculin test using an interferon-gamma release assay as a reference standard. Pediatr Infect Dis J. 2011; 30: 426-8.

137. Papay P, Eser A, Winkler S, Frantal S, Primas C, Miehsler W, et al. Factors impacting the results of interferon-gamma release assay and tuberculin skin test in routine screening for latent tuberculosis in patients with inflammatory bowel diseases. Inflamm Bowel Dis. 2011; 17: 84-90.

138. Ramos JM, Masiá M, Rodríguez JC, López C, Padilla S, Robledano C, et al. Negative effect of immunosuppressive therapy in the performance of the QuantiFERON gold in-tube test in patients with immune-mediated inflammatory diseases. Clin Exp Med. 2013; 13: 177-86.

139. Ramos JM, Robledano C, Masiá M, Belda S, Padilla S, Rodríguez JC, et al. Contribution of interferon gamma release assays testing to the diagnosis of latent tuberculosis infection in HIV-infected patients: a comparison of QuantiFERON-TB Gold In Tube, T-SPOT.TB and tuberculin skin test. BMC Infect Dis. 2012; 12: 169.

140. Sauzullo I, Mengoni F, Scrivo R, Valesini G, Potenza C, Skroza N, Marocco R, et al. Evaluation of QuantiFERON-TB Gold In-Tube in human immunodeficiency virus infection and in patient candidates for anti-tumour necrosis factor-alpha treatment. Int J Tuberc Lung Dis. 2010; 14: 834-40.

141. Takahashi H, Shigehara K, Yamamoto M, Suzuki C, Naishiro Y, Tamura Y, et al. Interferon gamma assay for detecting latent tuberculosis infection in rheumatoid arthritis patients during infliximab administration. Rheumatol Int. 2007; 27: 1143-8.

142. Vassilopoulos D, Tsikrika S, Hatzara C, Podia V, Kandili A, Stamoulis N, et al. Comparison of two gamma interferon release assays and tuberculin skin testing for tuberculosis screening in a cohort of patients with rheumatic diseases starting anti-tumor necrosis factor therapy. Clin Vaccine Immunol. 2011; 18: 2102-8.

143. Hoffmann M, Tsinalis D, Vernazza P, Fierz W, Binet I, et al. Assessment of an Interferon-gamma release assay for the diagnosis of latent tuberculosis infection in haemodialysis patient. Swiss Med Wkly. 2010; 140: 286-92.

144. Mariette X, Baron G, Tubach F, Lioté F, Combe B, Miceli-Richard C, et al. Influence of replacing tuberculin skin test with ex vivo interferon gamma release assays on decision to administer prophylactic antituberculosis antibiotics before anti-TNF therapy. Ann Rheum Dis. 2012; 71: 1783-90.

145. Ponce de Leon D, Acevedo-Vasquez E, Alvizuri S, Gutierrez C, Cucho M, Alfaro J, et al. Comparison of an interferon-gamma assay with tuberculin skin testing for detection of tuberculosis (TB) infection in patients with rheumatoid arthritis in a TB-endemic population. J Rheumatol. 2008; 35: 776-81.

146. Scrivo R, Sauzullo I, Mengoni F, Iaiani G, Vestri AR, Priori R, et al. Serial interferon-gamma release assays for screening and monitoring of tuberculosis infection during treatment with biologic agents. Clin Rheumatol. 2012; 31: 1567-75.

147. Cho H, Kim YW, Suh CH, Jung JY, Um YJ, Jung JH, et al. Concordance between the tuberculin skin test and interferon gamma release assay (IGRA) for diagnosing latent tuberculosis infection in patients with systemic lupus erythematosus and patient characteristics associated with an indeterminate IGRA. Lupus. 2016; 25: 1341-8.

148. Kurti Z, Lovasz BD, Gecse KB, Balint A, Farkas K, Morocza-Szabo A, et al. Tuberculin skin test and Quantiferon in BCG vaccinated, immunosuppressed patients with moderate-to-severe inflammatory bowel disease. J Gastrointestin Liver Dis. 2015; 24: 467-72.

149. Kussen GM, Dalla-Costa LM, Rossoni A, Raboni SM. Interferon-gamma release assay versus tuberculin skin test for latent tuberculosis infection among HIV patients in Brazil. Braz J Infect Dis. 2016; 20: 69-75.

150. Palomar R, Arias Guillén M, Robledo C, Agüero R, Agüero J, Rodríguez C, et al. Detection of latent tuberculosis infection in peritoneal dialysis patients: new methods. Nefrologia. 2011; 31: 169-73.

151. Anibarro L, Trigo M, Feijoo D, Rios M, Palomares L, Pena A, et al. Tuberculin skin test and interferon-gamma release assay show better correlation after the tuberculin 'window period' in tuberculosis contacts. Scand J Infect Dis. 2011; 43: 424-9.

152. Diel R, Loddenkemper R, Meywald-Walter K, Niemann S, Nienhaus A. Predictive value of a whole blood IFN-gamma assay for the development of active tuberculosis disease after recent infection with Mycobacterium tuberculosis. Am J Respir Crit Care Med. 2008; 177: 1164-70.

153. Ferreira TF, da Fonseca Silva Matsouka P, dos Santas AM, de Jesus Mendes Caldas. Diagnosis of latent Mycobacterium tuberculosis infection: tuberculin test versus interferon-gamma release. Rev Soc Bras Med Trop. 2015; 48: 724-30.

154. Nienhaus A, Schablon A, Le Bacle C, Siano B. Evaluation of the interferon-gamma release assay in healthcare workers. Int Arch Occup Environ Health. 2008; 81: 295-300.

155. Mínguez S, Latorre I, Mateo L, Lacoma A, Diaz J, Olivé A, et al. Interferon-gamma release assays in the detection of latent tuberculosis infection in patients with inflammatory arthritis scheduled for anti-tumour necrosis factor treatment. Clin Rheumatol. 2012; 31: 785-94.

156. Moon SM, Lee SO, Choi SH, Kim YS, Woo JH, Yoon DH, et al. Comparison of the QuantiFERON-TB Gold In-Tube test with the tuberculin skin test for detecting latent tuberculosis infection prior to hematopoietic stem cell transplantation. Transpl Infect Dis. 2013; 15: 104-9.

157. Talati NJ, Seybold U, Humphrey B, Aina A, Tapia J, Weinfurter P, et al. Poor concordance between interferon-gamma release assays and tuberculin skin tests in diagnosis of latent tuberculosis infection among HIV-infected individuals. BMC Infect Dis. 2009; 9: 15.
