# Supplementary material for: Interferon-gamma release assay for the diagnosis of latent tuberculosis infection: A latent-class analysis
Source: PLoS One. 2017 Nov 28;12(11):e0188631. doi: 10.1371/journal.pone.0188631 (PMC5705142; doi:10.1371/journal.pone.0188631)
Supplement: S2 Table — *Results are for immune-competent adults. BCG, Bacillus Calmette-Guérin; CrI, credible interval; QFT-GIT, QuantiFERON-TB Gold In Tube; TB, tuberculosis; TST, tuberculin skin test. (PDF) [file pone.0188631.s005.pdf]

S2 Table. Sensitivity of results to exclusion of studies deemed to be of high risk of bias.

Parameter	Diagnostic test	Base case median (95% CrI)*	Exclusion of studies of high risk of bias median (95% CrI)*
Sensitivity (%)	QFT-GIT	52 (50 - 53)	53 (51 - 54)
	TST	84 (82 - 85)	83 (82 - 85)
Specificity (%)	QFT-GIT (non-BCG)	97 (96 - 97)	97 (96 - 98)
	QFT-GIT (BCG)	93 (92 - 94)	93 (92 - 94)
	TST (non-BCG)	100 (99 - 100)	100 (99 - 100)
	TST (BCG)	79 (76 - 82)	76 (74 - 79)

*Results are for immune-competent adults.

BCG, Bacillus Calmette-Guérin; CrI, credible interval; QFT-GIT, QuantiFERON-TB Gold In Tube; TB, tuberculosis; TST, tuberculin skin test.
