# Supplementary material for: Interferon-gamma release assay for the diagnosis of latent tuberculosis infection: A latent-class analysis
Source: PLoS One. 2017 Nov 28;12(11):e0188631. doi: 10.1371/journal.pone.0188631 (PMC5705142; doi:10.1371/journal.pone.0188631)
Supplement: S3 Table — *Results are for immune-competent adults. BCG, Bacillus Calmette-Guérin; CrI, credible interval; QFT-GIT, QuantiFERON-TB Gold In Tube; TB, tuberculosis; TST, tuberculin skin test. (PDF) [file pone.0188631.s006.pdf]

S3 Table. Sensitivity of results to prior distributions.

Parameter	Prior distribution	Model estimates* median (95% CrI)	Prior distribution	Model estimates* median (95% CrI)	Prior distribution	Model estimates* median (95% CrI)
Sensitivity QFT-GIT (%)	Beta(15,5.3) (base case)	52 (50 - 53)	Uniform(0.55,0.93)	55 (55 - 55)	Uniform(0.5,1)	52 (50 - 53)
Sensitivity TST (%)	Beta(14.6,4.6) (base case)	84 (82 - 85)	Uniform(0.57,0.95)	83 (82 - 84)	Uniform(0.5,1)	84 (82 - 85)
Specificity QFT-GIT (non-BCG) (%)	Beta(64,3.7) (base case)	97 (96 - 97)	Uniform(0.89,1)	97 (96 - 98)	Uniform(0.5,1)	97 (96 - 97)
Specificity QFT-GIT (BCG) (%)	Beta(64,3.7) (base case)	93 (92 - 94)	Uniform(0.89,1)	93 (92 - 95)	Uniform(0.5,1)	93 (92 - 94)
Specificity TST (non-BCG) (%)	Beta(9.9,2.9) (base case)	100 (99 - 100)	Uniform(0.55,1)	100 (100 - 100)	Uniform(0.5,1)	100 (99 - 100)
Specificity TST (BCG) (%)	Beta(9.9,2.9) (base case)	79 (76 - 82)	Uniform(0.55,1)	73 (71 - 75)	Uniform(0.5,1)	79 (76 - 82)

*Results are for immune-competent adults.

BCG, Bacillus Calmette-Guérin; CrI, credible interval; QFT-GIT, QuantiFERON-TB Gold In Tube; TB, tuberculosis; TST, tuberculin skin test.
